# Genetic variants and their association with phenotypic resistance to bedaquiline in *Mycobacterium tuberculosis*: a systematic review and individual isolate data analysis

**DOI:** 10.1016/s2666-5247(21)00175-0

**Published:** 2021-08-31

**Authors:** Nabila Ismail, Emmanuel Rivière, Jason Limberis, Stella Huo, John Z Metcalfe, Rob M Warren, Annelies Van Rie

**Affiliations:** South African Medical Research Council Centre for Tuberculosis Research and Department of Science and Innovation - National Research Foundation Centre of Excellence for Biomedical Tuberculosis Research, Division of Molecular Biology and Human Genetics, Faculty of Medicine and Health Sciences, Stellenbosch University, Cape Town, South Africa (N Ismail PhD, Prof R M Warren PhD); Family Medicine and Population Health, Faculty of Medicine, University of Antwerp, Antwerp, Belgium (E Rivière MSc, Prof A Van Rie PhD); Division of Experimental Medicine, University of California, San Francisco, San Francisco, CA, USA (J Limberis PhD); Division of Pulmonary and Critical Care Medicine, Zuckerberg San Francisco General Hospital and Trauma Centre, University of California, San Francisco, San Francisco, CA, USA (S Huo MSc, Prof J Z Metcalfe PhD)

## Abstract

**Background:**

Bedaquiline is a crucial drug for control of rifampicin-resistant tuberculosis. Molecular drug resistance assays could facilitate effective use of bedaquiline and surveillance of drug resistance emergence. To facilitate molecular assay development, we aimed to identify genomic markers of bedaquiline resistance.

**Methods:**

In this systematic review and individual isolate analysis, we searched Europe PubMed Central and Scopus for studies published from the inception of each database until Oct 19, 2020, that assessed genotypic and phenotypic bedaquiline resistance in clinical or non-clinical *Mycobacterium tuberculosis* isolates. All studies reporting on the assessment of variants in the four genes of interest (*Rv0678*, *atpE*, *pepQ*, and *Rv1979c*) and phenotypic bedaquiline data in both clinical and non-clinical samples were included. We collated individual isolate data from eligible studies to assess the association between genomic variants with phenotypic bedaquiline resistance, using a standardised method endorsed by WHO. Risk of bias of the extracted data was independently assessed by two authors using the Quality Assessment of Diagnostic Accuracy Studies tool for clinical studies and Systematic Review Center for Laboratory Animal Experimentation tool for animal studies. The primary outcome was to identify mutations associated with resistance in four genes of interest (*Rv0678*, *atpE*, *pepQ*, and *Rv1979c*); for each genomic variant, the odds ratio (OR), 95% CI, and p value were calculated to identify resistance markers associated with bedaquiline resistance. This study is registered with PROSPERO, CRD42020221498.

**Findings:**

Of 1367 studies identified, 41 published between 2007 and 2020 were eligible for inclusion. We extracted data on 1708 isolates: 1569 (91·9%) clinical isolates and 139 (8·1%) non-clinical isolates. We identified 237 unique variants in *Rv0678*, 14 in *atpE*, 28 in *pepQ,* and 11 in *Rv1979c*. Most clinical isolates with a single variant reported in *Rv0678* (229 [79%] of 287 variants), *atpE* (14 [88%] of 16 variants), *pepQ* (32 [100%] of 32 variants), or *Rv1979c* (115 [98%] of 119 variants) were phenotypically susceptible to bedaquiline. Except for the *atpE* 187G→C (OR ∞, [95% CI 13·28–∞]; p<0·0001) and *Rv0678* 138_139insG (OR 6·91 [95% CI 1·16–47·38]; p=0·016) variants, phenotypic–genotypic associations were not significant (p≥0·05) for any single variant in *Rv0678*, *atpE*, *pepQ,* and *Rv1979c*.

**Interpretation:**

Absence of clear genotypic–phenotypic associations for bedaquiline complicates the development of molecular drug susceptibility tests. A concerted global effort is urgently needed to assess the genotypic and phenotypic drug susceptibility of *M tuberculosis* isolates, especially in patients who have received unsuccessful bedaquiline-containing regimens. Treatment regimens should be designed to prevent emergence of bedaquiline resistance and phenotypic drug susceptibility tests should be used to guide and monitor treatment.

**Funding:**

Research Foundation Flanders, South African Medical Research Council, Department of Science and Innovation - National Research Foundation, National Institute of Health Institute of Allergy and Infectious Diseases, and Doris Duke Charitable Foundation.

## Introduction

Half a million people were diagosed with rifampicin-resistant tuberculosis in 2019.^1^ The expedited approval of bedaquiline in 2012 allowed for swift access by people with multidrug-resistant tuberculosis; it also enabled the development of all-oral multidrug-resistant tuberculosis treatment regimens and improved survival of patients with multidrug-resistant tuberculosis.^2–4^ However, the expedited approval of bedaquiline meant that concurrent implementation of validated genomic or phenotypic drug susceptibility tests (DST) was not possible. To date, many countries still do not have phenotypic DST capacity for bedaquiline.^5–7^

Development of an accurate molecular assay requires a strong understanding of the genetic correlates of bedaquiline resistance. Bedaquiline targets subunit C of *Mycobacterium tuberculosis* ATP synthase—encoded by the *atpE* gene—inhibiting energy production.^8^ Laboratory experiments have shown an association between *atpE* variants and bedaquiline resistance.^9^ Clinical studies found that resistance was also associated with variants in the *Rv0678* (*mmpR*) gene, which encodes a repressor protein regulating efflux pump expression via the *mmpS5-mmpL5* operon, thereby implicating a drug efflux mechanism in bedaquiline resistance.^10,11^ In 2016, *pepQ* (*rv2535c*) and *Rv1979c* were identified as genes that might be associated with bedaquiline resistance.^12,13^ Mutations in *pepQ* (encoding a putative Xaa-Pro amino-peptidase) and *Rv1979c* (encoding a putative permease) have been implicated in bedaquiline resistance, but the underlying mechanisms remain unknown.^14,15^

To facilitate the development of a molecular bedaquiline resistance diagnostic tool, we did a systematic literature review and pooled individual isolate data analysis to assess the association between phenotypic resistance and variants in the *Rv0678*, *atpE*, *pepQ,* and *Rv1979c* genes.

## Methods

### Search strategy and selection criteria

In this systematic review and individual isolate analysis, we included studies reporting phenotypic DST or minimal inhibitory concentration (MIC) and assessment of variants in the four genes of interest (*Rv0678*, *atpE*, *pepQ,* and *Rv1979c*) in both clinical and non-clinical *M tuberculosis* isolates. We excluded conference abstracts and book chapters or studies not reporting original data. We searched Europe PubMed Central and Scopus for articles published from inception of each database to Oct 19, 2020, using the terms (“bedaquiline” OR “sirturo” OR “TMC207” OR “R207910”) AND (“tuberculosis” OR “TB”) AND (“MIC” OR “MICs”) OR (“minimum” AND “inhibitory” AND concentration*) OR resist* OR susceptib*) AND (muta* OR “genetic” OR “genome”: OR sequenc*) without language or date restrictions. When information was missing authors were contacted for clarification (eg, authors were contacted to confirm whether genotyping of *atpE* or *Rv0678* was done). A full search of the grey literature was not done, but WHO publications were eligible for inclusion. Two authors independently screened articles for eligibility. After removal of duplicate entries, article titles and abstracts were reviewed to exclude studies not related to *M tuberculosis* or bedaquiline; basic science research articles not focusing on phenotype–genotype association and studies reporting on general clinical and epidemiological topics of tuberculosis were also excluded. The full text of selected articles was reviewed to confirm eligibility. The reference lists of meta-analyses, review articles, and included manuscripts were searched (By ER) for eligible publications missed by the search.

### Data analysis

Variables extracted at the individual isolate level were geographical origin of isolation; isolate type (clinical, murine, or in vitro); bedaquiline exposure status; variants reported in *Rv0678*, *atpE*, *pepQ,* and *Rv1979c* or any additional gene as indicated by the author; variant type (single nucleotide polymorphism [SNP] and insertions or deletions); nucleotide and amino acid changes; presence of co-occurring variants; bedaquiline MIC; phenotypic DST; and sequencing method. Data on isolate lineage was not extracted because these were available for only a minority of isolates. When either nucleotide or amino acid change was not provided, missing data were inferred using Expasy,^16^ or through contact with the author. Data were extracted manually by one investigator (ER) and was checked by a second (AVR). Authors were contacted when information was missing. For isolates reported by multiple studies, only the first study was chosen unless the later study reported additional phenotypic or genotypic data.

Two investigators (ER and AVR) independently assessed the risk of bias and concern of applicability using the Quality Assessment of Diagnostic Accuracy Studies tool for clinical studies (QUADAS-2) and Systematic Review Center for Laboratory Animal Experimentation tool for animal studies (SYRCLE; appendix 1 pp 3–7, 8–14).^17,18^ Quality was not assessed for the in-vitro studies because no comprehensive and standardised guidelines were available.

We classified isolates as phenotypically susceptible or resistant to bedaquiline according to the breakpoint concentrations of 1 μg/mL for mycobacteria growth indicator tube (MGIT), 0·25 μg/mL for 7H11,^19^ 0·25 μg/mL for 7H10,^20^ and 0·125 μg/mL for 7H9 broth microdilution formats, including microplate alamarBlue assay (MABA), resazurin microtiter assay (REMA) and Thermo Fisher Scientific microtiter plates.^21^ Phenotypic results were classified as indeterminate out of quality concern (silent mutations classified as resistant),^22^ or when the method was not specified.^23^ Classification agreement of isolates as phenotypically resistant or susceptible by different methods was investigated for isolates assessed by multiple methods. For isolates with information on at least the *atpE* and *Rv0678* genes, we described the MIC distribution of (1) wild-type isolates; (2) isolates with one or more variants in the *Rv0678* gene; (3) one or more variants in the *atpE* gene; and (4) variants in both *atpE* and *Rv0678*. MIC distributions of isolates with only *pepQ* or *Rv1979c* variants were not described due to scarce data.

We collated the genotypic and phenotypic data of individual isolates and applied the standardised method for interpreting the association between variants (exposure) and phenotypic resistance (outcome).^24^ Because of the large number of variants and the paucity of data per variant, we included clinical and non-clinical isolates.^25^ Isolates were excluded if variants were not reported for both *atpE* and *Rv0678* genes. Association with phenotypic resistance was investigated for variants that were observed at least once when occurring alone in phenotypically resistant isolates and for variants reported independently of the presence of co-occurring variants for phenotypically susceptible isolates. We also investigated the association of combinations of mutations observed more than once in phenotypically resistant isolates with bedaquiline resistance.

The primary outcome was to identify mutations associated with resistance in four genes of interest: *atpE*, *Rv0678*, *pepQ*, and *Rv1979c*. We also investigated the association with bedaquiline resistance for combinations of variants.

Odds ratios (OR) were used to evaluate the association of the genotypic and phenotypic data. 95% CIs and p values were calculated using the fisher test function in the stats package R (version 4.0.0). Except for non-sense mutations and frameshift mutations, p values were adjusted for false discovery rate using the Benjamini-Hochberg procedure. Associations with phenotypic resistance were considered significant when the p value was less than 0·05 and the OR CIs did not cross 1 (appendix 1 pp 2–3). This study is registered with PROSPERO, CRD42020221498.

### Role of the funding source

The funders of the study had no role in study design, data collection, data analysis, data interpretation, or writing of the report.

## Results

**Figure 1:**
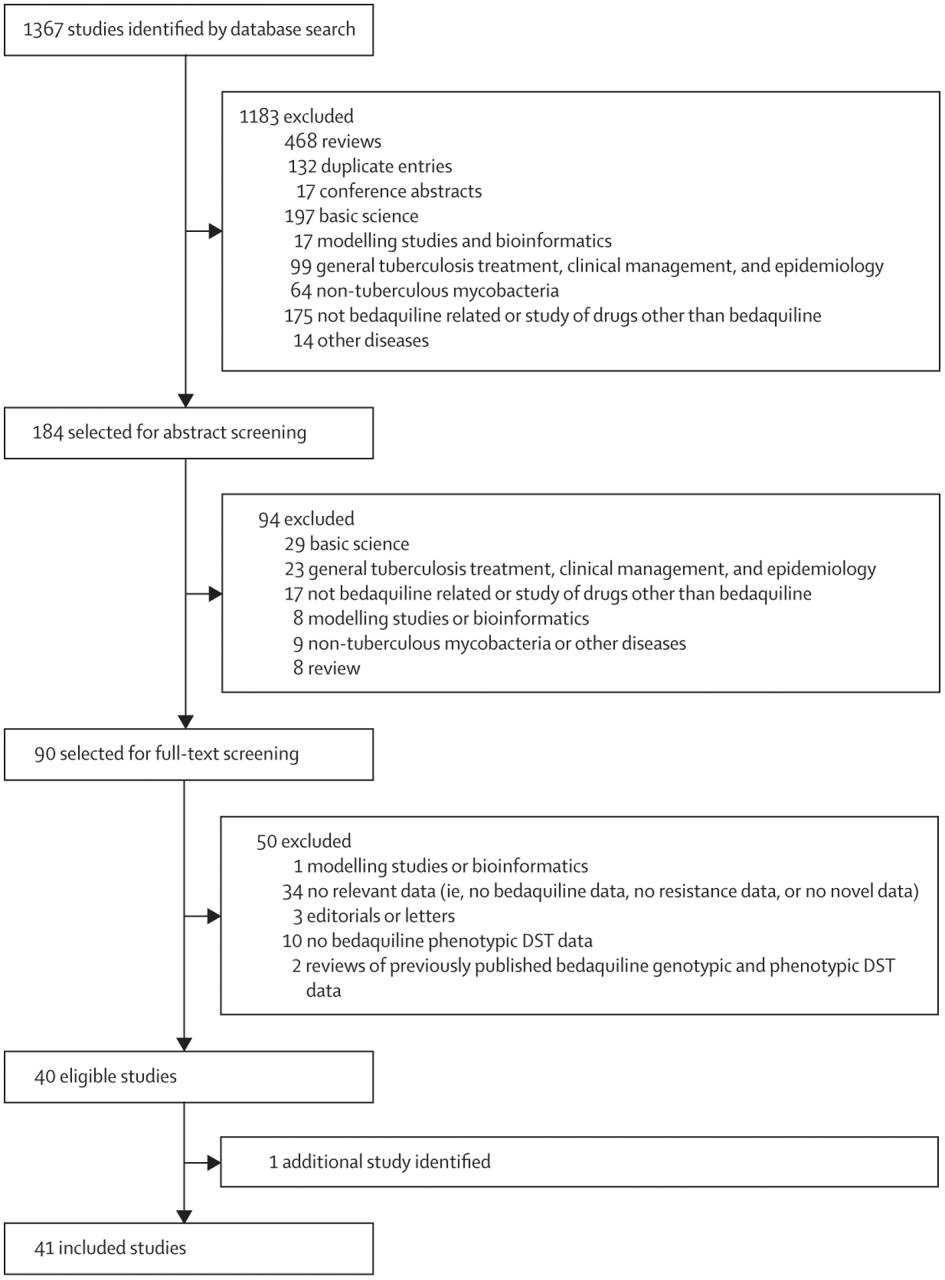
Study profile DST=drug susceptibility tests.

Of the 1367 identified studies, 40 (2·9%) were eligible for inclusion.^8,10,11,13,21–23,26–58^ Additionally, a technical report published by WHO was identified and included (figure 1).^19^ QUADAS-2 assessment of the clinical studies showed that the risk of bias was unclear for 19 (68%) of 28 studies^19,22,23,26,29,30,32,33,39,44,46–49,52,53,55,56,58^ due to a possible absence of masked interpretation of mutations; risk of bias was high for 11 studies^23,27,28,32,35,40,43,44,47,48,53^ that used a phenotypic DST method not approved by WHO (appendix 1 pp 3–7). According to the QUADAS-2 assessment, the level of concern regarding applicability was classified as high for six (21%) of the 28 clinical studies^23,35,45,48,49,51^ because they did not report on both *atpE* and *Rv0678* genes. SYRCLE assessment of the animal studies showed that all studies had an unclear risk of selection, performance, and detection bias, and a low risk of attrition, reporting, or other sources of bias (appendix 1 pp 8–14).

The review included data on 1708 *M tuberculosis* isolates: 1569 (91·9%) clinical and 139 (8·1%) non-clinical isolates. Of the 1569 clinical isolates (originating from 32 countries; appendix 1 p 15), 1198 (76·4%) were obtained from patients who were bedaquiline naive and 297 (18·9%) from patients who had been exposed to bedaquiline. No exposure data were available for the remaining 74 (4·7%) patients. Of the 139 non-clinical isolates, 113 (81%) were in-vitro manipulated samples and 26 (19%) were isolates from murine studies. To identify genomic variants, 19 (46%) of 41 studies used whole-genome sequencing, 14 (34%) used targeted sequencing, and five (12%) studies used both (confirmed whole-genome sequencing results with targeted sequencing); three studies did not specify the sequencing technique used (table 1). Four (10%) studies reported on variants in *atpE* only, six (15%) reported on *Rv0678* only, 11 (27%) on *Rv0678* and *atpE*, nine (22%) on *Rv0678*, *atpE and pepQ*, and 11 (27%) studies reported on all four genes of interests (*Rv0678*, *atpE*, *pepQ,* and *Rv1979c*; table 1). Two (5%) studies reported on *Rv0677c*,^26,32^ and one (2%) study on *atpB*.^43^ 15 studies (933 samples) used MGIT, 13 (913 samples) used 7H11 media, eight (70 samples) used MABA, eight (158 samples) used REMA, three (423 samples) used Thermo Fisher Scientific microtiter plates, three (42 samples) used 7H10 media, and one (35 samples) used 7H9 media with tetrazolium chloride to determine the bedaquiline MIC; seven studies used two methods and two studies used three methods for all isolates (table 1). Overall, 1383 (82·7%) of the 1672 isolates were classified as phenotypically susceptible, and 289 (17·3%) as resistant to bedaquiline. Agreement in classification of isolates was high between MGIT and Thermo Fisher microtiter plate (393 [99%] of 396 isolates had the same classification), and between MGIT and 7H11 (445 [97%] of 459 isolates had the same classification; appendix 1 p 16). Estimates for agreement between other methods was imprecise due to the low number of samples.

**Figure 2:**
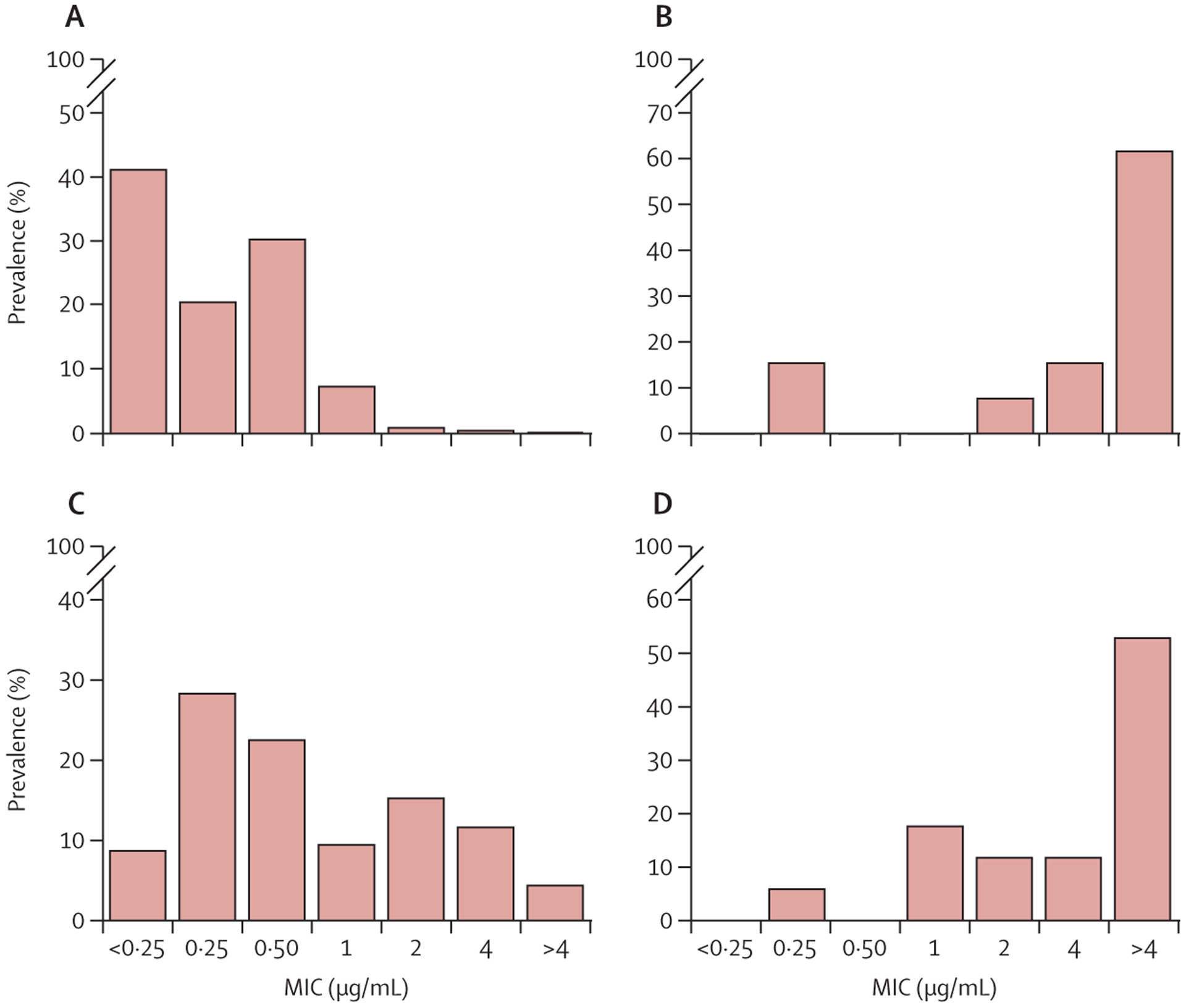
MGIT MIC distribution Only isolates with information on both *atpE* and *Rv0678* genes were included. Isolates for which the reported MIC could not be reported as one of the concentrations in this figure were excluded. (A) MGIT MIC distribution of wild-type samples (652 isolates). (B) MGIT MIC distribution of isolates with one or more *atpE* variants and wild-type *Rv0678* (13 isolates). (C) MGIT MIC distribution of isolates with one or more *Rv0678* variants and wild-type *atpE* (138 isolates). (D) MGIT MIC distribution of isolates with one or more *atpE* variants and one or more *Rv0678* variants (17 isolates). MGIT=mycobacteria growth indicator tube. MIC=minimal inhibitory concentration.

31 (76%) of the studies reported on variants in *atpE* and *Rv0678* (table 1). Of the 1178 isolates analysed in these studies, 819 (69·5%) were wild type, of which 807 (99%) were clinical isolates. 401 (62%) of the 652 wild-type isolates had a MGIT MIC of 0·25 μg/mL or less and 648 (99%) of 652 isolates had an MIC of 1 μg/mL or less. Only four (1%) of the 652 wild-type isolates were phenotypically resistant on MGIT (MIC >1 μg/mL; figure 2A). MIC distributions of wild-type isolates on other platforms were similar to MGIT (appendix 1 pp 17–18).

**Figure 3:**
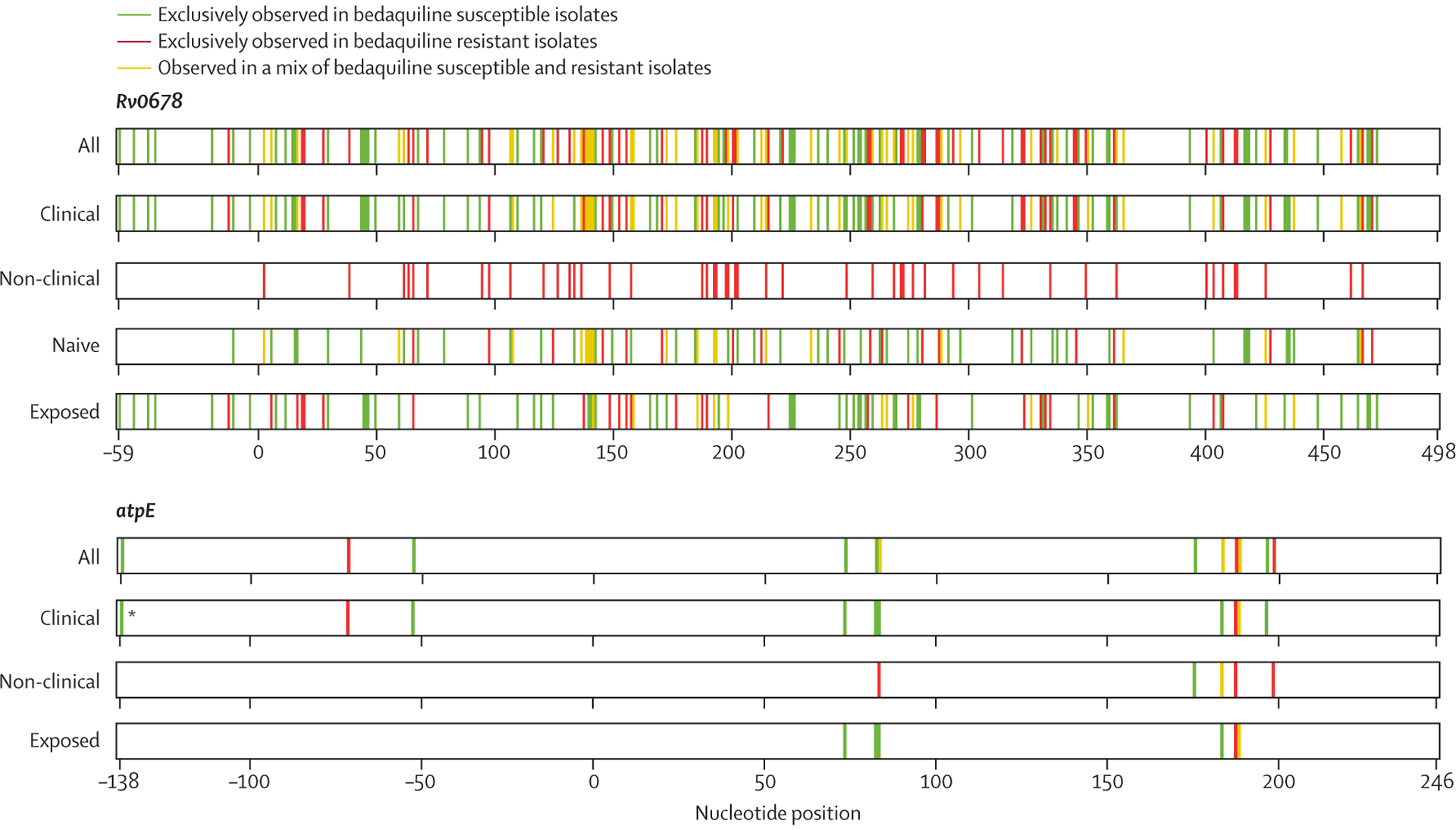
Observed variants across the *Rv0678* and *atpE* genes Position of observed variants across the *Rv0678* and *atpE* genes in all samples and in samples stratified by origin (clinical and non-clinical) and bedaquiline exposure status (exposed and naive) are shown. *Only *atpE* variant observed in an isolate from a patient who was bedaquiline treatment naive.

37 (90%) studies reported on *Rv0678* variants in 1653 isolates. 1214 (73·4%) of these isolates were *Rv0678* wild type and 439 (26·6%) contained one or more *Rv0678* variants (table 1; appendix 2). 386 (88%) of the 439 samples with a mutation in the *Rv0678* gene were clinical samples. Variants occurred along the entire 498 base-pair coding region of the *Rv0678* gene and in the 85 base-pair intergenic region between the *Rv0678* and *Rv0677c* genes (figure 3; appendix 1 p 19). Overall, 237 unique *Rv0678* variants were identified at 209 different positions of the *Rv0678* gene: 152 (64%) unique SNPs (including ten silent mutations) and 85 (36%) unique insertions or deletions. 142 (60%) of the 237 unique variants were reported only once. 395 (90%) of the 439 isolates with mutations had either a single (320 [73%]) or multiple (75 [17%]) *Rv0678* variants with no variants reported in the other genes of interest. 23 (5%) of 439 isolates contained both *Rv0678* and *atpE* variants, and 21 (5%) isolates had co-occurring *Rv1979c* variants. 79 (95%) of the 83 isolates with multiple variants in the *Rv0678* gene were clinical isolates, of which 57 (72%) were retrieved from patients who had previously received bedaquiline. The MIC of isolates that had one or more *Rv0678* variants but were *atpE* wild type ranged from less than 0·25 to more than 4 μg/mL, and 49 (34%) of these 145 isolates were phenotypically resistant on MGIT (figure 2C). MIC distributions of isolates with *Rv0678* variants on other platforms were similar to MGIT (appendix 1 pp 17–18). Of the 439 samples with *Rv0678* variants, 386 (88%) were clinical isolates, 198 (51%) were retrieved from patients who were bedaquiline naive and 174 (45%) from patients who had previously received bedaquiline; bedaquiline exposure was unknown for the remaining 14 (4%) isolates. 229 (80%) of 287 clinical isolates with any type of *Rv0678* variants (143 had SNPs and 144 had insertions or deletions) but no variants in the other genes of interest were phenotypically susceptible isolates: 120 (83%) of 144 with insertions or deletions and 109 (76%) of 143 with SNPs.

35 (85%) studies reported on *atpE* variants in 1233 isolates, of which 1145 (92·9%) were *atpE* wild type and 88 (7·1%) contained one or more *atpE* variants (table 1; appendix 2). Overall, 14 unique variants were reported at 10 distinct positions in the 246 base-pair-long coding region (figure 3, appendix 1 p 20). One (7%) SNP was synonymous, one (7%) was non-sense, and 12 (86%) were missense. No insertions or deletions were reported. Of the 14 unique *atpE* variants reported, seven (50%) were only reported once. Possible hotspot regions were located at positions 82 and 83 and 183–198. In addition, three mutations were identified in the 200 base-pair upstream region of the *atpE* gene. 65 (74%) of 88 isolates with *atpE* variants had either a single (n=62) or multiple (n=3) variants in this gene but none in the other genes of interest; the remaining 23 (26%) isolates had variants in both *atpE* and *Rv0678*, whereas none had co-occurring variants in *pepQ* or *Rv1979c*. 12 (86%) of 14 isolates carrying only *atpE* variants and 14 (78%) of 18 isolates carrying *atpE* and *Rv0678* variants were phenotypically resistant on MGIT (figure 2B, D). MIC distributions of isolates with *atpE* variants and a combination of *atpE* and *Rv0678* variants on other platforms are reported in the appendix 1 (pp 17–18), but are difficult to compare with MGIT due to the scarcity of data. Of the 26 clinical isolates containing one or more variants in the *atpE* gene, three (12%) were retrieved from patients who were bedaquiline naive, 18 (69%) from patients who were bedaquiline exposed, and five (19%) had an unknown exposure status. Of the 16 clinical isolates with an isolated *atpE* variant and no other mutations, 14 (88%) were phenotypically susceptible to bedaquiline.

20 (49%) studies reported on 1061 isolates with variants in *pepQ* (table 1; appendix 2), of which 1022 (96·3%) were wild type and 39 (3·7%) contained one of 28 unique variants in the *pepQ* gene or one of two variants upstream in the *pepQ* gene (appendix 1 pp 21–22). All 32 clinical isolates with *pepQ* variants were phenotypically susceptible; three (43%) of the seven murine isolates with a *pepQ* variant were phenotypically resistant to bedaquiline (appendix 2).

11 (27%) studies reported on variants in *Rv1979c* (table 1; appendix 2). Overall, 18 unique *Rv1979c* variants were reported in 140 clinical isolates (appendix 1 pp 23–24). 115 (97%) of 119 isolates without co-occurring variants in other genes of interest were phenotypically susceptible to bedaquiline (appendix 2).

Two (5%) studies reported on variants in the *mmpS5-mmpL5* genes.^26,32^ Two variants were reported in clinical isolates (appendix 2). The isolate containing the *mmpL5* 1030G→C variant was phenotypically resistant to bedaquiline; the isolate containing the *mmpL5* 1804T→C variant was phenotypically susceptible to bedaquiline. In the only study reporting on *atpB* variants,^43^ three variants without co-occurring variants in other genes of interest were reported upstream of *atpE*: −53G→A, −72T→C, and −138T→C. Only the −72T→C variant was reported in a phenotypically resistant clinical isolate.

Of the 1708 isolates included in our study, 36 (2·1%) were excluded from the statistical analysis of the genotype–phenotype association due to quality concerns on the phenotypic DST and 529 (31·0%) were excluded because of an absence of data on both *atpE* and *Rv0678*. Of the 1143 included isolates, 659 (57·7%) were wild type, 292 (25·5%) were phenotypically susceptible and contained one or more variants in the genes of interest, and 192 (16·8%) were phenotypically resistant. 1071 (93·7%) of 1143 isolates were clinical isolates, of which 724 (67·6%) were from patients who were bedaquiline naive and 277 (25·9%) from patients who were bedaquiline exposed. Only one insertion mutation in *Rv0678* (138_139insG) was associated with phenotypic resistance (OR 6·91 [95% CI 1·16–47·38]; p=0·016). There was no evidence of association with resistance (p≥0·05) for the other 59 insertions or deletions and any of the 102 assessed SNPs in the *Rv0678* gene (table 2; appendix 3). The only variant in the *atpE* gene associated with resistance was the 187G→C mutation (OR ∞ [13·28–∞]; p<0·0001), which was reported in ten in-vitro isolates and one clinical isolate (table 3). There was no evidence of association with phenotypic resistance for any of the 27 *pepQ* variants and 17 *Rv1979c* variants (p≥0·05; table 3).

16 unique combinations of variants were reported more than once: eight dual *Rv0678* variants, one dual *atpE* variant, three combinations of variants in *atpE* and *Rv0678*, three combinations of variants in *Rv0678* and *Rv1979c*, and one combination of three *Rv0678* variants. All four combinations containing a variant in *atpE* were associated with resistance (OR ≥24·7, p<0·05; appendix 1 p 25). The dual *atpE* combination contained two variants (82G→A and 183G→T) that were both reported in susceptible isolates when occurring alone. One combination contained the 187G→C *atpE* variant, which was associated with resistance when occurring alone, together with the 141_142insC variant in *Rv0678*, which was reported as a single variant in 13 phenotypically susceptible and four resistant isolates. One combination contained a deletion in *Rv0678* with the 83A→C variant in *atpE*, which also occurred alone in a resistant isolate. One combination contained the 188C→T *atpE* and the 425T→G *Rv0678* variants, both of which were only reported in phenotypically susceptible isolates when occurring alone. Of the eight dual *Rv0678* variant combinations, two were associated with resistance (OR∞ [95% CI 1·51–∞]; p=0·024; appendix 1 p 25). Both contained the 141_142insC variant, once in combination with the 138_139insG variant—which were shown to be associated with resistance in isolation—and once in combination with the 322A→G variant—which has not yet been reported alone. The other seven combinations of *Rv0678* variants, which consisted of two variants that had either been reported in isolation solely in susceptible isolates or had been reported in both susceptible and resistant isolates, were not associated with resistance (p≥0·05). Two of the three combinations of a variant in *Rv0678* and *Rv1979c* were associated with resistance (OR ≥24·8, p<0·024; appendix 1 p 25). Both contained the 1226G→A variant in *Rv1979c*, which had been reported alone in two phenotypically resistant and 37 phenotypically susceptible isolates.

Treatment outcome data were available for 56 patients (appendix 4), of whom nine (16%) had a variant present in *atpE* or *Rv0678* at the start of treatment. 36 (80%) of 45 patients with wild-type *Rv0678* and *atpE* at baseline acquired a variant during bedaquiline treatment. Treatment was classified as successful in 26 (46%) of 56 patients after culture conversion, but 17 (30%) patients did not have culture conversion; of these 17 patients, eight (47%) died. Three (5%) of 56 patients relapsed, of whom one (33%) died, and treatment was ongoing in eight (14%) patients. Two (4%) of 56 patients were lost to follow-up. Nine (35%) of 26 patients whose treatment was classified as successful had wild-type *atpE* and *Rv0678* at the end of treatment. The other 47 (84%) of 56 patients for whom treatment outcome data were available had at least one variant in one of the genes of interest at the end of treatment.

## Discussion

Our results show that 8 years after US Food and Drug Administration approval of bedaquiline for multidrug-resistant tuberculosis treatment,^2^ the association of genotypic variants with phenotypic resistance or clinical outcomes remains unclear due to scarce data and study heterogeneity. By summarising 13 years of data, we generated the most exhaustive catalogue to date: 14 unique variants in *atpE*, 237 in *Rv0678*, 28 in *pepQ*, and 11 in *Rv1979c*. Results of our systematic literature review confirmed that variants in the *atpE* gene result in high level resistance,^31^ but the evidence originates predominantly from in vitro and animal experiments, with few documented clinical cases. Our results show that variants in the *Rv0678* gene are numerous and scattered throughout the gene, with most of the SNPs, insertions, and deletions occurring in phenotypically susceptible clinical isolates. Although *pepQ* and *Rv1979c* have been hypothesised to play a role in the development of bedaquiline resistance,^12,13^ the data we collated on these genes showed that it is unlikely that they play an important role in resistance.

Our analysis is the first to statistically evaluate the association between variants in *atpE*, *Rv0678*, *pepQ*, and *Rv1979c* with phenotypic resistance. Using a standard methodology,^24^ two single variants (*atpE* 187G→C and *Rv0678* 138_139insG) were associated with resistance. However, this knowledge will not contribute substantially to clinical care because the *atpE* 187G→C variant was only reported once and the *Rv0678* 138_139insG variant three times in clinical isolates over the past 13 years. None of the other single variants assessed in *atpE*, *Rv0678*, *pepQ*, or *Rv1979c* were associated with phenotypic resistance (p≥0·05), likely due to scarce data. Eight combinations of variants were associated with resistance. One contained the *atpE* 187G→C variant and one the *Rv0678* 138_139insG variant, confirming their association with resistance. The only dual *atpE* combination contained two variants (82G→A and 183G→T) only reported in susceptible isolates when occurring in isolation. The *Rv1979c* 1226G→A variant, which occurred in 37 susceptible isolates and two resistant isolates and was associated with resistance when occurring in combination with two different *Rv0678* variants that were not associated with resistance when occurring in isolation. These findings highlight the difficulty of translating the phenotype of a variant when it occurs in isolation to a co-occurring variant. An updated literature search done during the review process of this Article on March 27, 2021, yielded one additional study that would have been eligible for inclusion in our systematic review and analysis.^59^ This study reported one new variant (*Rv0678* 110A→V), with all other variants already included in our dataset. Data from this study were not extracted because they would not affect the results and interpretation of our study.

Our review extends the knowledge on bedaquiline resistance by increasing the number of articles reviewed from 18–22^14,15,60^ to 41, by stratifying information by bedaquiline exposure status, and by summarising the effect of genetic variants on treatment outcome. Despite these strengths, several limitations should be noted. To collect as much data as possible, we included both clinical and non-clinical studies. Although it is broadly accepted that in-vivo antibiotic resistance can be replicated in vitro, this assumption has yet to be proven for bedaquiline specifically.^25^ Likewise, we included data from multiple clinical studies of different designs to increase the amount of data. Regarding phenotypic DST, multiple methodologies were used, but only MGIT, 7H11, and Thermo Fisher microdilution plates have been validated.^21^ Although no provisional breakpoints have been endorsed for MABA and REMA, these methods were used for a minority of samples and inter-phenotypic DST agreement with other methods was high. Because not all studies investigated or reported all genes of interest, the sample size of the genotype–phenotype analysis was reduced by only including samples with data on both *atpE* and *Rv0678*. This restriction was not applied to the *pepQ* and *Rv1979c* genes due to scare data. Most variants were only reported in a small number of isolates, many co-occurred with other variants, and minor variants were not always assessed, complicating the assessment of the genotype–phenotype association. Variants in genes that might compensate for loss of function by another variant, as has been shown for *mmpS5-mmpL5* variants co-occurring with *Rv0678* variants,^14^ were rarely reported. Clofazimine exposure status was only reported in 19 (46%) of 41 studies. This impeded full description of previous selection pressure on *Rv0678* because clofazimine can result in cross resistance with bedaquiline.^34^ Our aim to assess the effect of resistance conferred by specific variants on treatment outcome was limited by scarce data. Finally, multiple studies were at high risk of bias due to incomplete description of the phenotypic DST and genotyping methods used. Future studies should be designed and reported according to international guidelines for diagnostic accuracy studies.^17,61^

In summary, our findings show that our current knowledge on the genomic basis of bedaquiline resistance is insufficient to develop a rapid molecular assay. To advance our knowledge on the phenotypic–genotypic association for bedaquiline, a concerted effort is needed to report comprehensive genotypic (preferably whole-genome sequencing) and phenotypic (using standardised methodologies) data together with treatment outcome information, especially in people who experience treatment failure. Alternative approaches to determine the genotypic–phenotypic association should be explored, and multidrug-resistant tuberculosis treatment regimens should be designed to protect bedaquiline; phenotypic DST should be used to guide and monitor treatment of patients suffering from multidrug-resistant tuberculosis.

## Supplementary Material

1

2

3

4

## Figures and Tables

**Table 1:** Study characteristics

	Phenotyping method	Clofazimine DST	Sample type	Bedaquiline exposure	Genotyping method	*atpE*	*Rv0678*	pepQ	*Rv1979c*	Number of isolates
Ismail et al (2018)^37^	MGIT	Yes	In vitro	NA	WGS	Yes	Yes	Yes	Yes	9
Bloemberg et al (2015)^28^	MGIT	Yes	Clinical	Exposed	Targeted	Yes	Yes	NR	NR	3
Hoffman et al (2016)^35^	MGIT	Yes	Clinical	Exposed	WGS	NR	Yes	NR	NR	1
Xu et al (2017)^56^	MABA	Yes	Clinical	Naive	WGS and targeted	Yes	Yes	Yes	Yes	6
Veziris et al (2017)^52^	7H11	No	Clinical	Mix	Targeted	Yes	Yes	NR	NR	5
Villellas et al (2017)^53^	7H11	Yes	Clinical	Naive	Targeted	Yes	Yes	Yes	NR	347
Zimenkov et al (2017)^58^*	7H11	No	Clinical	Mix	Targeted	Yes	Yes	Yes	Yes	85
Peretokina et al (2020)^47^*	MGIT and 7H11	No	Clinical	Mix	Targeted	Yes	Yes	Yes	Yes	344
Pang et al (2017)^46^	MABA	Yes	Clinical	Naive	Targeted	Yes	Yes	Yes	NR	5
Ismail et al (2018)^39^	MGIT and TF plate	Yes	Clinical	Mix	WGS	Yes	Yes	Yes	Yes	391
Martinez et al (2018)^43^	REMA	Yes	Clinical	Naive	WGS	Yes	Yes	Yes	NR	56
Ghodousi et al (2019)^33^	MGIT and 7H11	Yes	Clinical	Mix	WGS	Yes	Yes	Yes	Yes	19
Almeida et al (2016)^13^	7H11	Yes	Murine	NA	WGS and targeted	Yes	Yes	Yes	NR	4
Torrea et al (2015)^51^	MGIT and 7H11	No	Clinical	Naive	Targeted	NR	Yes	NR	NR	98
Yang et al (2018)^22^	MTT	No	Clinical	Naive	Targeted	Yes	Yes	NR	NR	35
Ismail et al (2019)^38^	MGIT	Yes	In vitro	NA	WGS	Yes	Yes	Yes	Yes	12
Ismail et al (2019)^36^	7H10	No	In vitro	NA	WGS	Yes	Yes	NR	NR	6
Ghajavand et al (2019)^32^	MABA	No	Clinical	Naive	WGS	Yes	Yes	Yes	Yes	24
de Vos et al (2019)^30^	MGIT	No	Clinical	Mix	WGS	Yes	Yes	NR	NR	7
Polsfuss et al (2019)^48^	MGIT and REMA	Yes	Clinical	Mix	WGS	NR	Yes	NR	NR	5
Rancoita et al (2018)^49^	7H11 and REMA	Yes	Clinical	NR	WGS	NR	Yes	NR	NR	4
Xu et al (2017)^55^	MABA	Yes	In vitro and clinical	Naive	Targeted	Yes	Yes	Yes	NR	7
Andries et al (2014)^11^†	REMA	Yes	In vitro and murine	NA	WGS and targeted	Yes	Yes	NR	NR	22
Koul et al (2007)^41^†	REMA, MABA, and 7H10	No	In vitro	NA	Targeted	Yes	NR	NR	NR	2
Xu et al (2019)^54^	MABA	No	Murine	NA	WGS and targeted	Yes	Yes	Yes	NR	18
Klopper et al (2020)^40^	MGIT	No	Clinical	Naive	WGS	Yes	Yes	NR	NR	1
Kaniga et al (2020)^21^	MGIT, 7H11, and TF plate	No	In vitro	NA	NR	Yes	Yes	NR	NR	5
Nimmo et al (2020)^44^	7H11	No	Clinical	Mix	WGS	NR	Yes	NR	NR	19
Andres et al (2020)^26^	MGIT	Yes	Clinical	Mix	WGS	Yes	Yes	Yes	Yes	20
Tantry et al (2017)^50^	MABA	No	In vitro	NA	WGS	Yes	NR	NR	NR	2
Conradie et al (2020)^29^	MGIT	Yes	Clinical	Exposed	WGS	Yes	Yes	NR	NR	1
WHO (2018)^19^	MGIT, and 7H11	Yes	Clinical	Mix	NR	Yes	Yes	NR	NR	53
Battaglia et al (2020)^27^	REMA	No	Clinical	Naive	WGS	Yes	Yes	Yes	NR	51
Nimmo et al (2020)^45^	7H11	No	Clinical	Mix	WGS	Yes	Yes	Yes	Yes	7
Liu et al (2020)^42^	TF plate	No	Clinical	Mix	Targeted	Yes	Yes	Yes	Yes	27
Degiacomi et al (2020)^31^	REMA	No	In vitro	NA	WGS	Yes	Yes	Yes	NR	14
Yang et al (2020)^57^	MABA	No	Clinical	Naive	Targeted	Yes	Yes	Yes	NR	6
Yoshiyama et al (2020)^23^	NR	No	Clinical	Exposed	NR	NR	Yes	NR	NR	1
Huitric et al (2010)^10^	7H10	No	In vitro	NA	Targeted	Yes	NR	NR	NR	34
Hartkoorn et al (2014)^34^	REMA	Yes	In vitro	NA	WGS and targeted	Yes	Yes	NR	NR	6
Segala et al (2012)^8^	7H11	No	In vitro	NA	Targeted	Yes	NR	NR	NR	18
Total	··	··	··	··	··	35	37	20	11	1708‡

DST=drug susceptibility test. MABA=Microplate Alamar Blue Assay. MGIT=Mycobacterial Growth Incubator Tubes. MTT=2,3-diphenyl-5-(2-thienyl)-tetrazolium chloride. NA=not applicable. NR=not reported. REMA=Resazurin Microtiter plate Assay. TF=ThermoFisher. WGS=whole genome sequencing.

* Studies shared 70 isolates.

† Studies shared two isolates.

‡ Excluding 72 shared isolates.

**Table 2:** Association between insertions, deletions, and single nucleotide polymorphisms in the Rv0678 gene and phenotypic resistance

	Phenotypic DST results						Statistical association between phenotype and genetic variant*	
	All		Clinical		Non-clinical		OR estimate (95% CI)	p value†
	R	S	R	S	R	S		
**Insertions or deletions**								
198_199insG	2	0	2	0	0	0	∞ (0·93–∞)	0·028
212delC, 139_141insTG, 145–147indel, 16_17delGG, 172_173insIS6110, 18_19delGG, 19delG, and 330delA	1‡	0	1‡	0	0	0	∞ (0·13–∞)	0·17
259_260insG, 272_273insIS6110, 334_335insIS6110, 349_350insIS6110, 38_39insA, 65_66insIS6110, and 94_95insIS6110	1‡	0	0	0	1‡	0	∞ (0·13–∞)	0·17
138_139insG	4	3	4	3	0	0	6·91 (1·16–47·38)	0·016
193delG	2	5	0	5	2	0	2·03 (0·19–12·53)	0·33
141_142insC	4	13	4	13	0	0	1·68 (0·39–5·51)	0·32
192_193insG	3	14	3	14	0	0	1·10 (0·20–3·99)	0·75
274_275insA	1	5	1	5	0	0	1·00 (0·02–8·97)	1·00
192delG and 288delC	1‡	6‡	1‡	6‡	0	0	0·83 (0·02–6·89)	1·00
138_140insGA	0	1	0	1	0	0	0·00 (0·00–195·64)	1·00
107delG, 138_140insGG, 140_141insG, 140_141insG, 142_143delCT, 142_143insC, 142delC, 15DelG, 176_177delCG, 176_178insGC, 192_194insGG, 193_194insG, 214delC, 262_263insA, 274_283delTATTTCCGGT, 288delG, 318_320insCG, 335delC, 43_44insA, 437_438insT, 457delG, and 46indel	0	1‡	0	1‡	0	0	0·00 (0·00–192·59)	1·00
140_141insC, 291_292insA, 29delG, 359_360insG, 464_465insC, 465_466insC, 136_137insG, and 198delG	0	2‡	0	2‡	0	0	0·00 (0·00–26·43)	1·00
133_134insTG, 139_140insG, 184_185insC, and 435delT	0	3‡	0	3‡	0	0	0·00 (0·00–12·02)	1·00
434delT	0	4	0	4	0	0	0·00 (0·00–7·52)	1·00
16delG	0	5	0	5	0	0	0·00 (0·00–5·42)	0·60
418_419insG	0	9	0	9	0	0	0·00 (0·00–2·51)	0·37
Total	33	120	24	120	9	0		
**SNPs (including non-sense mutations)**								
189C→A	3	0	0	0	3	0	∞ (2·06–∞)	0·38
97A→G	2	0	1	0	1	0	∞ (0·94–∞)	0·45
152A→C, 158C→G, and 287G→A	2‡	0	2‡	0	0	0	∞ (0·93–∞)	0·45
400C→T (non-sense)	2	0	0	0	2	0	∞ (0·93–∞)	0·028
403C→G and 281G→A	2‡	0	0	0	2‡	0	∞ (0·93–∞)	0·45
280C→T and 361G→A	1‡	0	1‡	0	0	0	∞ (0·13–∞)	0·73
148C→T and 214C→T	1‡	0	0	0	1‡	0	∞ (0·13–∞)	0·73
276T→A (non-sense)	1	0	0	0	1	0	∞ (0·13–∞)	0·17
263A→G, 155C→T, 257C→T, 286C→T, 215G→A, 326G→C, 124T→C, 323T→C, 332T→A, and 437T→C	1‡	0	1‡	0	0	0	∞ (0·13–∞)	0·73
271A→C, 413A→G, 65A→T, 120G→C, 197G→A, 131T→C, and 407T→C	1‡	0	0	0	1‡	0	∞ (0·13–∞)	0·73
61G→T (non-sense)	1	0	0	0	1	0	∞ (0·13–∞)	0·17
15T→7C	2	1	2	1	0	0	10·05 (0·52–591·28)	0·73
5G→T	2	1	2	1	0	0	10·00 (0·52–588·18)	0·73
2T→C	1	1	1	1	0	0	5·03 (0·06–393·65)	1·00
265C→T (silent)	1	1	1	1	0	0	4·97 (0·06–389·55)	0·31
202A→G, 403C→T	1‡	1‡	0	1‡	1‡	0	4·97 (0·06–389·55)	1·00
107C→T, 158C→T, 176C→T, 185C→T, 296C→T, 193G→A, and 59T→G	1‡	1‡	1‡	1‡	0	0	4·97 (0·06–389·55)	1·00
337G→A and 350T→G	1‡	2‡	1‡	2‡	0	0	2·48 (0·04–47·88)	1·00
136T→C and 248T→C	0	1‡	0	1‡	0	0	0·00 (0·00–193·60)	1·00
225C→T (silent)	0	1	0	1	0	0	0·00 (0·00–192·59)	1·00
11A→C, 14A→G, 165A→C, 331A→G, 67A→G, 11C→A, 53C→A, 220C→G, 251C→T, 268C→T, 279C→A, 406C→G, 109G→A, 149G→C, 194G→C, 196G→T, 245G→A, 259G→A, 352G→A, 358G→A, 393G→C, 417G→A, 421G→C, 61G→A, 20T→A, 240T→G, 254T→G, 274T→G, 416T→C, 437T→G, 469T→G, 59T→C, and 93T→G	0	1‡	0	1‡	0	0	0·00 (0·00–192·59)	1·00
425T→G	0	2	0	2	0	0	0·00 (0·00–27·13)	1·00
187A→G	0	2	0	2	0	0	0·00 (0·00–26·70)	1·00
106G→A and 365T→C	0	2‡	0	2‡	0	0	0·00 (0·00–26·56)	1·00
4A→T, 172A→C, 247C→G, 253G→T, 259G→C, 326G→T, 362G→A, 7G→A, 236T→C, and 278T→C	0	2‡	0	2‡	0	0	0·00 (0·00–26·43)	1·00
78T→G (non-sense)	0	2	0	2	0	0	0·00 (0·00–26·43)	1·00
798T→G (silent)	0	3	0	3	0	0	0·00 (0·00–12·02)	1·00
341T→C	0	4	0	4	0	0	0·00 (0·00–7·52)	1·00
67A→C and T25→4C	0	6‡	0	6‡	0	0	0·00 (0·00–4·22)	1·00
119T→C	0	7	0	7	0	0	0·00 (0·00–3·44)	1·00
Total	57	109	34	109	23	0	··	··

The variants that are observed only in resistant isolates are shown on top, with variants that are observed only in susceptible isolates are shown on the bottom of the table.

To be included studies had to report at a minimum on variants in both the *Rv0678* and *atpE* gene and co-occurring mutations could not be present in phenotypically resistant isolates. Del=deletion. DST=drug susceptibility test. Indel=insertion or deletion. Ins=insertion. OR=odds ratio. R=resistant. S=susceptible. SNP=single nucleotide polymorphism.

* Statistical analysis using the standardised method published by Miotto and colleagues.^24^

† p value adjusted for false discovery rate for all missense SNPs; p values were not adjusted for insertions, deletions, silent mutations, and non-sense mutations.

‡ n represents the number of strains reported with each of the unique variants listed.

**Table 3:** Association between variants in the *atpE*, *pepQ,* and *Rv1979c Rv0678* gene and phenotypic resistance

	Phenotypic DST results						Statistical association between phenotype and genetic variant*	
	All		Clinical		Non-clinical		OR estimate (95% CI)	p value†
	R	S	R	S	R	S		
**atpE**								
187G→C	11	0	1	0	10	0	∞ (13·28–∞)	<0·0001
83A→T	2	0	0	0	2	0	∞ (0·93–∞)	0·45
83A→C	1	0	0	0	1	0	∞ (0·13–∞)	0·73
72T→C	1	0	1	0	0	0	∞ (0·13–∞)	0·73
83A→G	3	1	0	1	3	0	15·57 (1·24–14·22)	0·45
183G→T	0	1	0	1	0	0	0·00 (0–199·86)	1·00
188C→T	0	1	0	1	0	0	0·00 (0–197·73)	1·00
196A→G, 53G→A, and 73G→A	0	1‡	0	1‡	0	0	0·00 (0–92·59)	1·00
183G→A (silent)	0	1	0	1	0	0	0·00 (0–92·59)	1·00
138T→C	0	3	0	3	0	0	0·00 (0–2·01)	1·00
G82G→A	0	4	0	4	0	0	0·00 (0–0·60)	1·00
Total	18	14	2	14	16	0	··	··
**pepQ**								
811delC and 131T→C	0	1‡	0	0	0	1‡	0·00 (0–230·84)	1·00
324A→G, 352A→G, 538A→G, 706A→G, 31C→T, 1114C→G, 206C→T, 269C→T, 371C→T, 433C→A, 7C→T, 914C→T, 12G→C, 1108G→A, 274G→A, 278G→A, 641T→C, 454G→A, 500G→T, and 640G→T	0	1‡	0	1‡	0	0	0·00 (0–30·84)	1·00
42delC	0	2	0	0	0	2	0·00 (0–1·67)	1·00
1021A→G, 407A→G, 233C→T, 20G→A, 347G→T, and 925G→A	0	2‡	0	2‡	0	0	0·00 (0–1·67)	1·00
Total	0	34	0	30	0	4	··	··
**Rv1979c**								
733A→C	1	0	1	0	0	0	∞ (0·19–∞)	0·73
1226G→A	2	37	2	37	0	0	0·39 (0·05–1·58)	1·00
114G→C	1	23	1	23	0	0	0·32 (0·01–0·01)	1·00
155A→C, 562C→T, 1216G→A, 724G→A, 1057T→G, 311T→C, and 824T→C	0	1‡	0	1‡	0	0	0·00 (0–291·36)	1·00
1403A→G, 20G→A, 187A→G, 1432C→G, and 520C→T	0	2‡	0	2‡	0	0	0·00 (0–40·09)	1·00
798G (silent)	0	2	0	2	0	0	0·00 (0–40·09)	1·00
114G→T or C	0	8	0	8	0	0	0·00 (0–4·42)	1·00
151T→A	0	14	0	14	0	0	0·00 (0–2·26)	1·00
857A→G	0	22	0	22	0	0	0·00 (0–1·36)	0·73
Total	3	121	3	121	0	0	··	··

The variants that are observed only in resistant isolates are shown on top, with variants that are observed only in susceptible isolates are shown on the bottom of the table.

To be included studies had to report at a minimum on variants in both the *Rv0678* and *atpE* genes and co-occurring mutations could not be present in phenotypically resistant isolates. Del=deletion. DST=drug susceptibility test. Ins=insertion. OR=odds ratio. R=resistant. S=susceptible.

* Statistical analysis using the standardised method published by Miotto and colleagues.^24^

† p value adjusted for false discovery rate for all missense single nucleotide polymorphism; p values were not adjusted for insertions, deletions, silent mutations, and non-sense mutations.

‡ n represents the number of strains reported with each of the unique variants listed.

## Research in context

### Evidence before this study

Multiple cases of unsuccessful treatment in patients receiving bedaquiline-containing regimens have been reported since bedaquiline was approved for the treatment of multidrug-resistant tuberculosis in 2012. Numerous variants in genes believed to be associated with bedaquiline resistance (*atpE*, *Rv0678*, *pepQ*, and *Rv1979c*) have been documented in in-vitro experiments, animal studies, and clinical *Mycobacterium tuberculosis* isolates. Three systematic reviews have collated data published between 2005 and 2018 on bedaquiline resistance. These systematic reviews were descriptive and did not aim to determine the statistical association between genomic variants and phenotypic bedaquiline resistance. Consequently, no genomic markers for bedaquiline resistance have been identified with confidence. To assess the association of genomic markers with bedaquiline resistance, we searched Europe PubMed Central and Scopus from the inception of each database to Oct 19, 2020, using the following search terms (“bedaquiline” OR “sirturo” OR “TMC207” OR “R207910”) AND (“tuberculosis” OR “TB”) AND (“MIC” OR “MICs”) OR (“minimum” AND “inhibitory” AND “concentration”*) OR resist* OR susceptib*) AND (mut* OR “genetic” OR “genome” OR sequenc*) without any language or date restrictions. An updated literature search done during the review process of this Article on March 27, 2021, using the same search criteria yielded one additional study which would have been eligible for inclusion in our study. However, this study was not included in this version of the systematic review because only one new variant, not already present in our dataset, was reported.

### Added value of this study

By increasing the number of included studies from 18–22 in previous reviews to 41, we present the most extensive catalogue of genomic variants in bedaquiline resistance associated genes (14 in *atpE*, 237 in *Rv0678*, 28 in *pepQ*, 11 in *Rv1979c*) and corresponding minimal inhibitory concentrations to date. We show that the highest number of unique variants occur in the *Rv0678* gene without any hotspot regions. Second, our review is the first to summarise data on genetic variants and treatment outcome, and to stratify results by type of study (clinical or non-clinical) and by exposure status to bedaquiline. We show that there is little evidence for the role of *atpE*, *pepQ,* and *Rv1979c* in clinical bedaquiline resistance. Third, we assessed the statistical association between the presence of genomic variants in the *atpE*, *Rv0678*, *pepQ,* and *Rv1979c* and phenotypic bedaquiline resistance. Using a standardised method, we show that only two single variants, *atpE* 187G→C and *Rv0678* 138_139insG, are associated with bedaquiline resistance. Furthermore, we identified eight unique combinations of mutations associated with resistance, three of which were a combination of variants in *atpE* and *Rv0678*, one dual variant in *atpE*, two a variant in *Rv1979c* and *Rv0678*, and two dual *Rv0678* variants. The statistical association with bedaquiline resistance for other mutations remains unknown.

### Implications of all the available evidence

The findings of a large (>200) number of genetic variants in *Rv0678* scattered across the gene, the occurrence of variants in both phenotypically susceptible and resistant *M tuberculosis* isolates and in isolates from patients who were bedaquiline naive and those who were previously exposed to bedaquiline, and the observation that only two genomic variants were statistically associated with bedaquiline resistance suggest that development of a rapid molecular drug susceptibility assay will be challenging. Consequently, to safeguard bedaquiline as an effective treatment option, treatment regimens should be carefully designed to avoid emergence of bedaquiline resistance, and phenotypic drug susceptibility testing methods should be used to guide and monitor treatment. A concerted research effort is needed to assess the genotypic and phenotypic drug susceptibility of *M tuberculosis* isolates, especially in patients in whom bedaquiline containing regimens were not successful.

## References

[1] WHO. Global tuberculosis report 2020. https://www.who.int/publications/i/item/9789240013131 (accessed June 3, 2020).

[2] Mahajan R Bedaquiline: first FDA-approved tuberculosis drug in 40 years. Int J Appl Basic Med Res 2013; 3: 1–2. 23776831 10.4103/2229-516X.112228 PMC3678673

[3] Schnippel K, Ndjeka N, Maartens G, Effect of bedaquiline on mortality in South African patients with drug-resistant tuberculosis: a retrospective cohort study. Lancet Respir Med 2018; 6: 699–706. 30001994 10.1016/S2213-2600(18)30235-2

[4] Bisson GP, Bastos M, Campbell JR, Mortality in adults with multidrug-resistant tuberculosis and HIV by antiretroviral therapy and tuberculosis drug use: an individual patient data meta-analysis. Lancet 2020; 396: 402–11. 32771107 10.1016/S0140-6736(20)31316-7 PMC8094110

[5] Mokrousov I, Akhmedova G, Polev D, Molchanov V, Vyazovaya A. Acquisition of bedaquiline resistance by extensively drug-resistant *Mycobacterium tuberculosis* strain of Central Asian Outbreak clade. Clin Microbiol Infect 2019; 25: 1295–97. 31229592 10.1016/j.cmi.2019.06.014

[6] Chawla K, Martinez E, Kumar A, Shenoy VP, Sintchenko V. Whole-genome sequencing reveals genetic signature of bedaquiline resistance in a clinical isolate of *Mycobacterium tuberculosis*. J Glob Antimicrob Resist 2018; 15: 103–04. 30248414 10.1016/j.jgar.2018.09.006

[7] Singh BK, Soneja M, Sharma R, Mutation in atpE and Rv0678 genes associated with bedaquline resistance among drug-resistant tuberculosis patients: a pilot study from a high-burden setting in Northern India. Int J Mycobacteriol 2020; 9: 212–15. 32474547 10.4103/ijmy.ijmy_30_20

[8] Segala E, Sougakoff W, Nevejans-Chauffour A, Jarlier V, Petrella S. New mutations in the mycobacterial ATP synthase: new insights into the binding of the diarylquinoline TMC207 to the ATP synthase C-ring structure. Antimicrob Agents Chemother 2012; 56: 2326–34. 22354303 10.1128/AAC.06154-11 PMC3346594

[9] Andries K, Verhasselt P, Guillemont J, A diarylquinoline drug active on the ATP synthase of *Mycobacterium tuberculosis*. Science 2005; 307: 223–27. 15591164 10.1126/science.1106753

[10] Huitric E, Verhasselt P, Koul A, Andries K, Hoffner S, Andersson DI. Rates and mechanisms of resistance development in *Mycobacterium tuberculosis* to a novel diarylquinoline ATP synthase inhibitor. Antimicrob Agents Chemother 2010; 54: 1022–28. 20038615 10.1128/AAC.01611-09 PMC2825986

[11] Andries K, Villellas C, Coeck N, Acquired resistance of *Mycobacterium tuberculosis* to bedaquiline. PLoS One 2014; 9: e102135. 25010492 10.1371/journal.pone.0102135 PMC4092087

[12] Zhang S, Chen J, Cui P, Shi W, Zhang W, Zhang Y. Identification of novel mutations associated with clofazimine resistance in *Mycobacterium tuberculosis*. J Antimicrob Chemother 2015; 70: 2507–10. 26045528 10.1093/jac/dkv150 PMC4539095

[13] Almeida D, Ioerger T, Tyagi S, Mutations in pepQ confer low-level resistance to bedaquiline and clofazimine in *Mycobacterium tuberculosis*. Antimicrob Agents Chemother 2016; 60: 4590–99. 27185800 10.1128/AAC.00753-16 PMC4958187

[14] Kadura S, King N, Nakhoul M, Systematic review of mutations associated with resistance to the new and repurposed *Mycobacterium tuberculosis* drugs bedaquiline, clofazimine, linezolid, delamanid and pretomanid. J Antimicrob Chemother 2020; 75: 2031–43. 32361756 10.1093/jac/dkaa136 PMC7825472

[15] Nieto Ramirez LM, Quintero Vargas K, Diaz G. Whole genome sequencing for the analysis of drug resistant strains of *Mycobacterium tuberculosis*: a systematic review for bedaquiline and delamanid. Antibiotics (Basel) 2020; 9: e133. 32209979 10.3390/antibiotics9030133 PMC7148535

[16] Gasteiger E, Gattiker A, Hoogland C, Ivanyi I, Appel RD, Bairoch A. ExPASy: the proteomics server for in-depth protein knowledge and analysis. Nucleic Acids Res 2003; 31: 3784–88. 12824418 10.1093/nar/gkg563 PMC168970

[17] Whiting PF, Rutjes AW, Westwood ME, QUADAS-2: a revised tool for the quality assessment of diagnostic accuracy studies. Ann Intern Med 2011; 155: 529–36. 22007046 10.7326/0003-4819-155-8-201110180-00009

[18] Hooijmans CR, Rovers MM, de Vries RB, Leenaars M, Ritskes-Hoitinga M, Langendam MW. SYRCLE’s risk of bias tool for animal studies. BMC Med Res Methodol 2014; 14: 43. 24667063 10.1186/1471-2288-14-43 PMC4230647

[19] WHO. Technical report on critical concentrations for TB drug susceptibility testing of medicines used in the treatment of drug-resistant TB. Geneva: World Health Organization, 2018.

[20] European Committee on Antimicrobial Susceptibility Testing. Breakpoint tables for interpretation of MICs and zone diameters Version 10.0. 2020. url: https://www.eucast.org/fileadmin/src/media/PDFs/EUCAST_files/Breakpoint_tables/v_10.0_Breakpoint_Tables.pdf (accessed Aug 18, 2021).

[21] Kaniga K, Aono A, Borroni E, Validation of bedaquiline phenotypic drug susceptibility testing methods and breakpoints: a multilaboratory, multicountry study. J Clin Microbiol 2020; 58: e01677–19. 31969421 10.1128/JCM.01677-19 PMC7098739

[22] Yang JS, Kim KJ, Choi H, Lee SH. Delamanid, bedaquiline, and linezolid minimum inhibitory concentration distributions and resistance-related gene mutations in multidrug-resistant and extensively drug-resistant tuberculosis in Korea. Ann Lab Med 2018; 38: 563–68. 30027700 10.3343/alm.2018.38.6.563 PMC6056398

[23] Yoshiyama T, Mitarai S, Takaki A, Multi-drug resistant tuberculosis with simultaneously acquired-drug resistance to bedaquiline and delamanid. Clin Infect Dis 2020; published online 7 30. 10.1093/cid/ciaa1064. 32730621

[24] Miotto P, Tessema B, Tagliani E, A standardised method for interpreting the association between mutations and phenotypic drug resistance in *Mycobacterium tuberculosis*. Eur Respir J 2017; 50: 1701354. 29284687 10.1183/13993003.01354-2017 PMC5898944

[25] Köser CU, Cirillo DM, Miotto P. How to optimally combine genotypic and phenotypic drug susceptibility testing methods for pyrazinamide. Antimicrob Agents Chemother 2020; 64: e01003–20. 32571824 10.1128/AAC.01003-20 PMC7449218

[26] Andres S, Merker M, Heyckendorf J, Bedaquiline-resistant tuberculosis: dark clouds on the horizon. Am J Respir Crit Care Med 2020; 201: 1564–68. 32053752 10.1164/rccm.201909-1819LE

[27] Battaglia S, Spitaleri A, Cabibbe AM, Characterization of genomic variants associated with resistance to bedaquiline and delamanid in naive *Mycobacterium tuberculosis* clinical strains. J Clin Microbiol 2020; 58: e01304–20. 32907992 10.1128/JCM.01304-20 PMC7587096

[28] Bloemberg GV, Keller PM, Stucki D, Acquired resistance to bedaquiline and delamanid in therapy for tuberculosis. N Engl J Med 2015; 373: 1986–88. 26559594 10.1056/NEJMc1505196 PMC4681277

[29] Conradie F, Diacon AH, Ngubane N, Treatment of highly drug-resistant pulmonary tuberculosis. N Engl J Med 2020; 382: 893–902. 32130813 10.1056/NEJMoa1901814 PMC6955640

[30] de Vos M, Ley SD, Wiggins KB, Bedaquiline microheteroresistance after cessation of tuberculosis treatment. N Engl J Med 2019; 380: 2178–80. 31141643 10.1056/NEJMc1815121 PMC6518951

[31] Degiacomi G, Sammartino JC, Sinigiani V, Marra P, Urbani A, Pasca MR. In vitro study of bedaquiline resistance in *Mycobacterium tuberculosis* multi-drug resistant clinical isolates. Front Microbiol 2020; 11: 559469. 33042066 10.3389/fmicb.2020.559469 PMC7527418

[32] Ghajavand H, Kargarpour Kamakoli M, Khanipour S, High prevalence of bedaquiline resistance in treatment-naive tuberculosis patients and verapamil effectiveness. Antimicrob Agents Chemother 2019; 63: e02530–18. 30602521 10.1128/AAC.02530-18 PMC6395892

[33] Ghodousi A, Rizvi AH, Baloch AQ, Acquisition of cross-resistance to bedaquiline and clofazimine following treatment for tuberculosis in Pakistan. Antimicrob Agents Chemother 2019; 63: e00915–19. 31262765 10.1128/AAC.00915-19 PMC6709449

[34] Hartkoorn RC, Uplekar S, Cole ST. Cross-resistance between clofazimine and bedaquiline through upregulation of MmpL5 in *Mycobacterium tuberculosis*. Antimicrob Agents Chemother 2014; 58: 2979–81. 24590481 10.1128/AAC.00037-14 PMC3993252

[35] Hoffmann H, Kohl TA, Hofmann-Thiel S, Delamanid and bedaquiline resistance in *Mycobacterium tuberculosis* ancestral Beijing genotype causing extensively drug-resistant tuberculosis in a Tibetan refugee. Am J Respir Crit Care Med 2016; 193: 337–40. 26829425 10.1164/rccm.201502-0372LE

[36] Ismail N, Ismail NA, Omar SV, Peters RPH. In vitro study of stepwise acquisition of *rv0678* and *atpE* mutations conferring bedaquiline resistance. Antimicrob Agents Chemother 2019; 63: e00292–19. 31138569 10.1128/AAC.00292-19 PMC6658778

[37] Ismail N, Omar SV, Ismail NA, Peters RPH. In vitro approaches for generation of *Mycobacterium tuberculosis* mutants resistant to bedaquiline, clofazimine or linezolid and identification of associated genetic variants. J Microbiol Methods 2018; 153: 1–9. 30165087 10.1016/j.mimet.2018.08.011

[38] Ismail N, Peters RPH, Ismail NA, Omar SV. Clofazimine exposure in vitro selects efflux pump mutants and bedaquiline resistance. Antimicrob Agents Chemother 2019; 63: e02141–18. 30642938 10.1128/AAC.02141-18 PMC6395909

[39] Ismail NA, Omar SV, Joseph L, Defining bedaquiline susceptibility, resistance, cross-resistance and associated genetic determinants: a retrospective cohort study. EBioMedicine 2018; 28: 136–42. 29337135 10.1016/j.ebiom.2018.01.005 PMC5835552

[40] Klopper M, Heupink TH, Hill-Cawthorne G, A landscape of genomic alterations at the root of a near-untreatable tuberculosis epidemic. BMC Med 2020; 18: 24. 32014024 10.1186/s12916-019-1487-2 PMC6998097

[41] Koul A, Dendouga N, Vergauwen K, Diarylquinolines target subunit c of mycobacterial ATP synthase. Nat Chem Biol 2007; 3: 323–24. 17496888 10.1038/nchembio884

[42] Liu Y, Gao M, Du J, Reduced susceptibility of *Mycobacterium tuberculosis* to bedaquiline during antituberculosis treatment and its correlation with clinical outcomes in China. Clin Infect Dis 2020; published online 7 15. 10.1093/cid/ciaa1002. 32667984

[43] Martinez E, Hennessy D, Jelfs P, Crighton T, Chen SC, Sintchenko V. Mutations associated with in vitro resistance to bedaquiline in *Mycobacterium tuberculosis* isolates in Australia. Tuberculosis 2018; 111: 31–34. 30029911 10.1016/j.tube.2018.04.007

[44] Nimmo C, Millard J, Brien K, Bedaquiline resistance in drug-resistant tuberculosis HIV co-infected patients. Eur Respir J 2020; 55: 1902383. 32060065 10.1183/13993003.02383-2019 PMC7270361

[45] Nimmo C, Millard J, van Dorp L, Population-level emergence of bedaquiline and clofazimine resistance-associated variants among patients with drug-resistant tuberculosis in southern Africa: a phenotypic and phylogenetic analysis. Lancet Microbe 2020; 1: e165–74. 32803174 10.1016/S2666-5247(20)30031-8 PMC7416634

[46] Pang Y, Zong Z, Huo F, In vitro drug susceptibility of bedaquiline, delamanid, linezolid, clofazimine, moxifloxacin, and gatifloxacin against extensively drug-resistant tuberculosis in Beijing, China. Antimicrob Agents Chemother 2017; 61: e00900–17. 28739779 10.1128/AAC.00900-17 PMC5610515

[47] Peretokina IV, Krylova LY, Antonova OV, Reduced susceptibility and resistance to bedaquiline in clinical *M tuberculosis* isolates. J Infect 2020; 80: 527–35. 31981638 10.1016/j.jinf.2020.01.007

[48] Polsfuss S, Hofmann-Thiel S, Merker M, Emergence of low-level delamanid and bedaquiline resistance during extremely drug-resistant tuberculosis treatment. Clin Infect Dis 2019; 69: 1229–31. 30933266 10.1093/cid/ciz074

[49] Rancoita PMV, Cugnata F, Gibertoni Cruz AL, Validating a 14-drug microtiter plate containing bedaquiline and delamanid for large-scale research susceptibility testing of *Mycobacterium tuberculosis*. Antimicrob Agents Chemother 2018; 62: e00344–18. 29941636 10.1128/AAC.00344-18 PMC6125532

[50] Tantry SJ, Markad SD, Shinde V, Discovery of Imidazo[1,2-a] pyridine ethers and squaramides as selective and potent inhibitors of mycobacterial adenosine triphosphate (ATP) synthesis. J Med Chem 2017; 60: 1379–99. 28075132 10.1021/acs.jmedchem.6b01358

[51] Torrea G, Coeck N, Desmaretz C, Bedaquiline susceptibility testing of *Mycobacterium tuberculosis* in an automated liquid culture system. J Antimicrob Chemother 2015; 70: 2300–05. 25977401 10.1093/jac/dkv117

[52] Veziris N, Bernard C, Guglielmetti L, Rapid emergence of *Mycobacterium tuberculosis* bedaquiline resistance: lessons to avoid repeating past errors. Eur Respir J 2017; 49: 1601719. 28182568 10.1183/13993003.01719-2016

[53] Villellas C, Coeck N, Meehan CJ, Unexpected high prevalence of resistance-associated *Rv0678* variants in MDR-TB patients without documented prior use of clofazimine or bedaquiline. J Antimicrob Chemother 2017; 72: 684–90. 28031270 10.1093/jac/dkw502 PMC5400087

[54] Xu J, Li SY, Almeida DV, Contribution of pretomanid to novel regimens containing bedaquiline with either linezolid or moxifloxacin and pyrazinamide in murine models of tuberculosis. Antimicrob Agents Chemother 2019; 63: e00021–19. 30833432 10.1128/AAC.00021-19 PMC6496099

[55] Xu J, Tasneen R, Peloquin CA, Verapamil increases the bioavailability and efficacy of bedaquiline but not clofazimine in a murine model of tuberculosis. Antimicrob Agents Chemother 2017; 62: e01692–17. 29038265 10.1128/AAC.01692-17 PMC5740328

[56] Xu J, Wang B, Hu M, Primary clofazimine and bedaquiline resistance among isolates from patients with multidrug-resistant tuberculosis. Antimicrob Agents Chemother 2017; 61: e00239–17. 28320727 10.1128/AAC.00239-17 PMC5444180

[57] Yang J, Pang Y, Zhang T, Molecular characteristics and in vitro susceptibility to bedaquiline of *Mycobacterium tuberculosis* isolates circulating in Shaanxi, China. Int J Infect Dis 2020; 99: 163–70. 32738481 10.1016/j.ijid.2020.07.044

[58] Zimenkov DV, Nosova EY, Kulagina EV, Examination of bedaquiline- and linezolid-resistant *Mycobacterium tuberculosis* isolates from the Moscow region. J Antimicrob Chemother 2017; 72: 1901–06. 28387862 10.1093/jac/dkx094

[59] Beckert P, Sanchez-Padilla E, Merker M, MDR *M tuberculosis* outbreak clone in Eswatini missed by Xpert has elevated bedaquiline resistance dated to the pre-treatment era. Genome Med 2020; 12: 104. 33239092 10.1186/s13073-020-00793-8 PMC7687760

[60] Ismail N, Omar SV, Ismail NA, Peters RPH. Collated data of mutation frequencies and associated genetic variants of bedaquiline, clofazimine and linezolid resistance in *Mycobacterium tuberculosis*. Data Brief 2018; 20: 1975–83. 30306102 10.1016/j.dib.2018.09.057 PMC6172430

[61] Cohen JF, Korevaar DA, Altman DG, STARD 2015 guidelines for reporting diagnostic accuracy studies: explanation and elaboration. BMJ Open 2016; 6: e012799. 10.1136/bmjopen-2016-012799 PMC5128957 28137831

