# Supplementary material for: Genetic variants and their association with phenotypic resistance to bedaquiline in Mycobacterium tuberculosis: a systematic review and individual isolate data analysis
Source: Lancet Microbe. Author manuscript; Available in PMC 2021 Nov 17. (PMC8597953; doi:10.1016/s2666-5247(21)00175-0)
Supplement: 1 [file NIHMS1753644-supplement-1.pdf]

THE LANCET
Microbe

Supplementary appendix 1

This appendix formed part of the original submission and has been peer reviewed. We post it as supplied by the authors.

Supplement to: Ismail N, Rivière E, Limberis J, et al. Genetic variants and their association with phenotypic resistance to bedaquiline in *Mycobacterium tuberculosis*: a systematic review and individual isolate data analysis. *Lancet Microbe* 2021; published online August 21. https://doi.org/10.1016/S2666-5247(21)00175-0.

Table of Contents

Supplementary data 1 – Extracted data 2
Supplementary data 2 – Statistical analysis output 2
Supplementary data 3 – Treatment outcome 2
Supplement 4 – Supplementary methods 2
Supplement 5 – QUADAS-2 3
Supplement 6 – SYRCLE 8
Supplement 7 – Geographical sample origin 15
Supplement 8 – pDST agreement 16
Supplement 9 – MIC 7H11 17
Supplement 10 – MIC Thermo Fisher microtiter plate 18
Supplement 11 – Rv0678 lollipop plot 19
Supplement 12 – atpE lollipop plot 20
Supplement 13 – pepQ heatmap 21
Supplement 14 – pepQ lollipop plot 22
Supplement 15 – Rv1979c heatmap 23
Supplement 16 – Rv1979c lollipop plot 24
Supplement 17 – Association between combinations of variants in the atpE, Rv0678, pepQ, and Rv1979c gene and phenotypic resistance 25

Supplementary data 1 – Extracted data

supplementary_data_1_extracted_data.xlsx

Supplementary data 2 – Statistical analysis output

supplementary_data_2_stats.xlsx

Supplementary data 3 – Treatment outcome

supplementary_data_3_outcome.xlsx

Supplement 4 – Supplementary methods

Data analysis

In order to statistically associate genomic variants with a bedaquiline phenotype, we performed an individual isolate analysis of pooled (collated) data, using the standard methodology published by Miotto et al.(1) In order to obtain the estimate of the odds ratio for each of the variants reported in the eligible studies, we first classified each sample as Resistant or Susceptible, based on the MIC value reported in the primary study. We classified isolates as phenotypically susceptible or resistant to bedaquiline according to the critical concentrations (CC) of 1 µg/ml for Mycobacteria Growth Indicator Tubes (MGIT), 0.25 µg/ml for 7H11,(2) 0.25 µg/ml for 7H10,(3) and 0.125 µg/ml for 7H9 broth microdilution formats including the microplate alamar Blue assay (MABA), resazurin microtiter assay (REMA) and Thermo Fisher Scientific microtiter plates.(4) Phenotypic results were classified as indeterminate out of quality concern (silent mutations classified as resistant) or when the method was not specified.(5, 6)

For each variant in the dataset, a 2x2 tables was constructed as follows:

total number (all studies combined) of phenotypically resistant eligible isolates containing the variant of interest	total number (all studies combined) of phenotypically resistant isolates not containing the variant of interest
total number (all studies combined) of phenotypically susceptible eligible isolates containing the variant of interest	total number (all studies combined) of phenotypically resistant isolates containing the variant

Using this 2x2 table, we calculated the effect estimate of interest (odds ratio), its 95% CI and p-value (Fisher's exact test). Following the method by Miotto et al. p-values were corrected using the False Discovery Rate (FDR) procedure, except for frameshift mutations and premature stop codons. These were treated as a special case, as the loss-of-function phenotype for these mutations can be predicted with confidence. In addition, we also calculated the sensitivity, specificity, positive predictive value, negative predictive value, accuracy, likelihood ratio estimates and their upper and lower bound. The complete output of the meta-analysis (including the numbers used to construct the 2x2 tables) is presented in the supplementary table 2.

An identical approach was followed to assess the association of combinations of mutations with phenotypic resistance. These combinations were also treated as a set of special cases for which the p-values were not corrected.

Confidence grading and interpretation

We applied the proposed confidence levels of grading mutations associated with phenotypic resistance as suggest by Miotto et al. with some minor changes. When the p-value was ≥0.05 the association was interpreted as 'no evidence of association' (instead of 'indeterminate' as proposed by Miotto et al.). Instead of classifying the associations as either 'high', 'moderate', 'minimal', or 'no association', we only report the odds ratio with corresponding 95% confidence interval and p-value without applying the classification, as only two variants were identified with a significant p-value and odds ratio lower bound >1 (*atpE* 187G>C, *Rv0678* 138_139insG).

The statistical method proposed by Miotto et al. and the corresponding interpretation is a very stringent method to identify new resistance markers in the absence of sufficient data. Due to the statistical interpretation, many mutations that are exclusively observed in resistant isolates are not considered as resistance markers. There are several ways one could re-classify the 'clinical' confidence in the association between phenotypic and genotypic resistance for these mutations. Variants reported in phenotypically resistant isolates by more than one study may be more likely to be truly conferring resistance than variants only reported by one study. The use of expert rules, as recently shown by Köser et al.,(7) is another way to re-classify confidence in resistance. However, the WHO has not yet recommended to apply these expert rules to bedaquiline resistance. Nevertheless, we report the phenotypic results and study references for all variants in the supplementary information (supplementary data 1: extracted data) to provide the opportunity of additional confidence grading.

Supplement 5 – QUADAS-2

Review Question

What is the strength of association between variants in the atpE, rv0678, pepQ and rv1979c gene and phenotypic resistance to bedaquiline?

Domain 1: Patient Selection

Risk of bias: Could the selection of patients/specimens have introduced bias?

Was a consecutive or random sample of patients or specimens enrolled?

'unclear' when the study did not report on patient selection, 'yes' when the study enrolled a consecutive or random sample, and 'no' for all other studies.

Was a case-control design avoided?

'no' when a case-control design was used, 'yes' when prospective or cross-sectional design was used.

Did the study avoid inappropriate exclusions?

'no' if the study excluded samples based on characteristics such as prior testing. 'yes' when no indications of inappropriate exclusion were noted.

Applicability: Are there concerns that the included patients and setting do not match the review question?

To statistically correlate genomic variants in bedaquiline resistance associated regions with bedaquiline resistance, we are interested in all samples reporting both relevant genomic and phenotypic data. Since our goal was to compile as much genotype-phenotype data as

possible, we do not expect any concerns of applicability concerning patient selection. We answered 'low concern' for all studies reporting on variants in at least *rv0678* and the *atpE* genes. When data was not reported on one of these genes, applicability concern was considered 'high'.

Domain 2: Index Test
Risk of Bias: Could the conduct or interpretation of the index test have introduced bias?
<u>Were the index test results interpreted with knowledge of the results of the reference standard?</u>
'yes' when genotyping and phenotyping were done in a blind manner; 'no' when genotyping and phenotyping were not done in a blind manner, and 'unclear' if not reported.
<u>If a threshold was used, was it prespecified?</u>
We applied our own threshold on phenotype data. We answered 'yes' for all studies.

Applicability: Are there concerns that the index test, its conduct, or its interpretation differ from the review question? Variations in test technology, execution, or interpretation may affect estimates of the diagnostic accuracy of a test.
Several methods are available for genotyping a clinical Mycobacterium tuberculosis isolate, such as whole genome sequencing and targeted (Sanger) sequencing. Several protocols and software packages are available for interpretation of genotypic data. When this was not reported, we answered 'unclear'. For all other studies, we answered 'low concern'.

Domain 3: Reference Standard
Risk of Bias: Could the reference standard, its conduct, or its interpretation have introduced bias?
<u>Is the reference standard likely to correctly classify the target condition?</u>
'yes' when the study used an WHO-recommended or an internationally recognized phenotypic DST method. 'unclear' when not enough information was provided regarding the pDST method. 'no' for not standard pDST methods.
<u>Were the reference standard results interpreted without knowledge of the results of the index test?</u>
'yes' when the index test was performed after the reference standard. 'unclear' when not reported (as was the case in most studies).

Applicability: Are there concerns that the target condition as defined by the reference standard does not match the question?
Bedaquiline is a new TB antibiotic. WHO and EUCAST have adopted standardized pDST protocols and provisional critical concentrations for platforms such as MGIT, 7H11, and 7H10. We answered 'low concern' for studies using one of these platforms. Other pDST methods are however being used, such as REMA, MTT, MABA, and broth microdilution on Thermo Fisher 96-wall plates. Since standardized protocols and critical concentrations do not yet exist for these reference methods, we answered 'unclear concern'.

Domain 4: Flow and Timing
Risk of Bias: Could the patient flow have introduced bias?
<u>Was there an appropriate interval between the index test and reference standard?</u>
This is not relevant for TB. 'yes' for all studies.
<u>Did all patients receive the same reference standard?</u>

‘yes’ when the same reference standard was used. ‘no’ when the samples were processed in different laboratories.

<u>Were all patients included in the analysis?</u>

‘yes’ for all studies.

		Risk of bias				Applicability		
**Ref**	**Study**	**Domain 1**	**Domain 2**	**Domain 3**	**Domain 4**	**Domain 1**	**Domain 2**	**Domain 3**
(8)	Bloemberg, 2015	🔴	🟢	🔴	🔴	🟢	🟢	🟡
(9)	Hoffman, 2016	🔴	🟢	🔴	🔴	🔴	🟢	🟢
(10)	Xu, 2018	🔴	🟡	🟢	🟢	🟢	🟢	🟢
(11)	Veziris, 2017	🟢	🟡	🟢	🟢	🟢	🟡	🟢
(12)	Villellas, 2016	🔴	🟡	🔴	🔴	🟢	🟡	🟢
(13)	Zimenkov; 2017	🟢	🟡	🟢	🟢	🟢	🟢	🟢
(14)	Pang, 2017	🟢	🟡	🟢	🟢	🟢	🟡	🟢
(15)	Ismail, 2018	🟢	🟡	🟢	🟢	🟢	🟢	🟢
(16)	Martinez, 2018	🟢	🟢	🔴	🟢	🟢	🟢	🟢
(17)	Ghodousi, 2019	🟢	🟡	🟢	🟢	🟢	🟢	🟢
(5)	Yang, 2018	🟢	🟡	🟢	🟢	🟢	🟡	🟡
(18)	Ghajavand, 2019	🟢	🟡	🔴	🟢	🟢	🟢	🟢
(19)	De Vos, 2020	🔴	🟡	🟢	🟢	🟢	🟢	🟢
(20)	Polsfuss, 2019	🟢	🟡	🔴	🟢	🔴	🟢	🟢
(21)	Rancoita, 2018	🟢	🟡	🟢	🟢	🔴	🟢	🟢
(22)	Xu, 2017	🔴	🟡	🟢	🟢	🟢	🟡	🟢
(23)	Klopper, 2020	🔴	🟢	🔴	🔴	🟢	🟡	🟢
(24)	Nimmo, 2020	🔴	🟡	🔴	🟢	🟢	🟢	🟢
(25)	Andres, 2020	🟢	🟡	🟢	🟢	🟢	🟢	🟢
(26)	Peretokina, 2020	🟢	🟡	🔴	🟢	🟢	🟢	🟢
(27)	Conradie, 2020	🔴	🟡	🟡	🟢	🟢	🟡	🟢
(28)	Battaglia, 2020	🔴	🟢	🔴	🔴	🟢	🟢	🟢
(29)	Torrea, 2015	🟡	🟢	🟢	🟢	🔴	🟢	🟢
(30)	WHO, 2018	🟡	🟡	🟡	🟡	🟡	🟡	🟡
(31)	Nimmo, 2020	🟢	🟢	🟢	🔴	🔴	🟢	🟢

(32)	Liu, 2020	🟡	🟢	🟢	🟢	🟢	🟢	🟢
(33)	Yang, 2020	🟢	🟢	🟢	🟢	🟢	🟢	🟢
(6)	Yoshiyama, 2020	🔴	🟡	🔴	🟢	🔴	🟡	🟡

Risk of bias

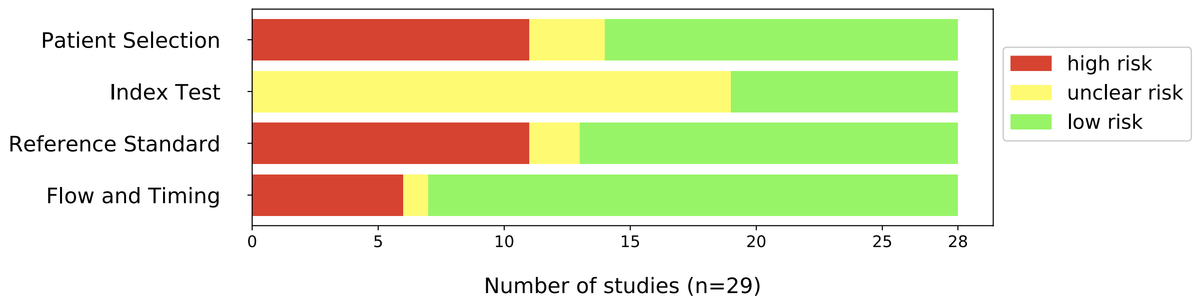

Applicability

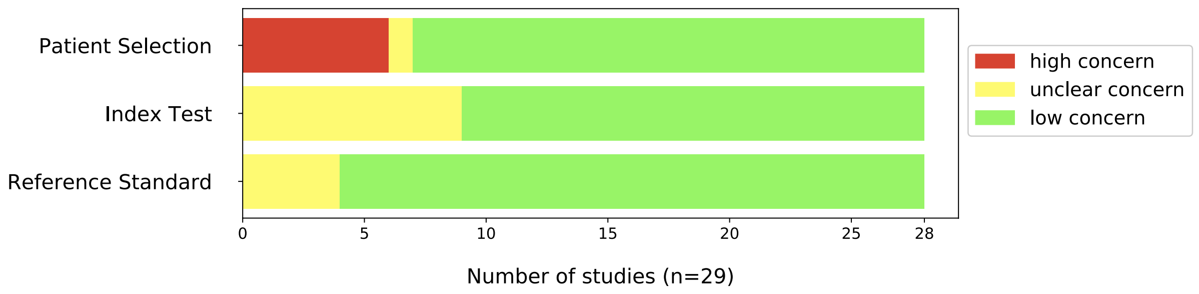

Supplement 6 – SYRCLE

1. Almeida 2016

1) Was the allocation sequence adequately generated and applied?

*Did the investigators describe a random component in the sequence generation process such as:

■ Referring to a random number table; Yes/No/Unclear

■ Using a computer random number generator.

Additional info:

2) Were the groups similar at baseline or were they adjusted for confounders in the analysis?

*Was the distribution of relevant baseline characteristics balanced for the intervention and control groups? Yes/No/Unclear

*If relevant, did the investigators adequately adjust for unequal distribution of some relevant baseline characteristics in the analysis? Yes/No/Unclear

*Was the timing of disease induction adequate? Yes/No/Unclear

3) Was the allocation to the different groups adequately concealed during?

*Could the investigator allocating the animals to intervention or control group not foresee assignment due to one of the following or equivalent methods? Yes/No/Unclear

4) Were the animals randomly housed during the experiment?

*Did the authors randomly place the cages or animals within the animal room/facility?

■ Animals were selected at random during outcome assessment (use signaling questions of entry 6). Yes/No/Unclear

*Is it unlikely that the outcome or the outcome measurement was influenced by not randomly housing the animals?

The animals from the various experimental groups live together in one cage/pasture (e.g., housing conditions are identical). Yes/No/Unclear

5) Were the caregivers and/or investigators blinded from knowledge which intervention each animal received during the experiment?

*Was blinding of caregivers and investigators ensured, and was it unlikely that their blinding could have been broken?

■ ID cards of individual animals, or cage/animal labels are coded and identical in appearance.

■ Sequentially numbered drug containers are identical in appearance. Yes/No/Unclear

■ The circumstances during the intervention are specified and similar in both groups (#).

■ Housing conditions of the animals during the experiment are randomized within the room (use criteria of entry 4).

6) Were animals selected at random for outcome assessment?

*Did the investigators randomly pick an animal during outcome assessment, or did they use a random component in the sequence generation for outcome assessment? Yes/No/Unclear

■ Referring to a random number table;

■ Using a computer random number generator;

■ Etc.

7) Was the outcome assessor blinded?

*Was blinding of the outcome assessor ensured, and was it unlikely that blinding could have been broken?	
■ Outcome assessment methods were the same in both groups.	Yes/No/Unclear
■ Animals were selected at random during outcome assessment (use signaling questions of entry 6).	
*Was the outcome assessor not blinded, but do review authors judge that the outcome is not likely to be influenced by lack of blinding? (e.g., mortality)	Yes/No/Unclear
**8) Were incomplete outcome data adequately addressed? (*)**	
*Were all animals included in the analysis?	Yes/No/Unclear
*Were the reasons for missing outcome data unlikely to be related to true outcome? (e.g., technical failure)	Yes/No/Unclear
*Are missing outcome data balanced in numbers across intervention groups, with similar reasons for missing data across groups?	Yes/No/Unclear
*Are missing outcome data imputed using appropriate methods?	Yes/No/Unclear
**9) Are reports of the study free of selective outcome reporting? (*)**	
*Was the study protocol available and were all of the study's pre-specified primary and secondary outcomes reported in the current manuscript?	Yes/No/Unclear
*Was the study protocol not available, but was it clear that the published report included all expected outcomes (i.e. comparing methods and results section)?	Yes/No/Unclear
**10) Was the study apparently free of other problems that could result in high risk of bias? (*)**	
*Was the study free of contamination (pooling drugs)?	Yes/No/Unclear
*Was the study free of inappropriate influence of funders?	Yes/No/Unclear
*Was the study free of unit of analysis errors?	Yes/No/Unclear
*Were design-specific risks of bias absent?	Yes/No/Unclear
*Were new animals added to the control and experimental groups to replace drop-outs from the original population?	Yes/No/Unclear

2. Andries 2014

1) Was the allocation sequence adequately generated and applied?

*Did the investigators describe a random component in the sequence generation process such as:

■ Referring to a random number table; Yes/No/Unclear

■ Using a computer random number generator.

Additional info:

2) Were the groups similar at baseline or were they adjusted for confounders in the analysis?

*Was the distribution of relevant baseline characteristics balanced for the intervention and control groups? Yes/No/Unclear

*If relevant, did the investigators adequately adjust for unequal distribution of some relevant baseline characteristics in the analysis? Yes/No/Unclear

*Was the timing of disease induction adequate? Yes/No/Unclear

3) Was the allocation to the different groups adequately concealed during?

*Could the investigator allocating the animals to intervention or control group not foresee assignment due to one of the following or equivalent methods? Yes/No/Unclear

4) Were the animals randomly housed during the experiment?

*Did the authors randomly place the cages or animals within the animal room/facility? Yes/No/Unclear

■ Animals were selected at random during outcome assessment (use signaling questions of entry 6).

*Is it unlikely that the outcome or the outcome measurement was influenced by not randomly housing the animals? Yes/No/Unclear

The animals from the various experimental groups live together in one cage/pasture (e.g., housing conditions are identical).

5) Were the caregivers and/or investigators blinded from knowledge which intervention each animal received during the experiment?

*Was blinding of caregivers and investigators ensured, and was it unlikely that their blinding could have been broken?

■ ID cards of individual animals, or cage/animal labels are coded and identical in appearance.

■ Sequentially numbered drug containers are identical in appearance. Yes/No/Unclear

■ The circumstances during the intervention are specified and similar in both groups (#).

■ Housing conditions of the animals during the experiment are randomized within the room (use criteria of entry 4).

6) Were animals selected at random for outcome assessment?

*Did the investigators randomly pick an animal during outcome assessment, or did they use a random component in the sequence generation for outcome assessment? Yes/No/Unclear

■ Referring to a random number table;

■ Using a computer random number generator;

■ Etc.

7) Was the outcome assessor blinded?

*Was blinding of the outcome assessor ensured, and was it unlikely that blinding could have been broken?	
■ Outcome assessment methods were the same in both groups.	Yes/No/Unclear
■ Animals were selected at random during outcome assessment (use signaling questions of entry 6).	
*Was the outcome assessor not blinded, but do review authors judge that the outcome is not likely to be influenced by lack of blinding? (e.g., mortality)	Yes/No/Unclear

8) Were incomplete outcome data adequately addressed? (*)

*Were all animals included in the analysis?	Yes/No/Unclear
*Were the reasons for missing outcome data unlikely to be related to true outcome? (e.g., technical failure)	Yes/No/Unclear
*Are missing outcome data balanced in numbers across intervention groups, with similar reasons for missing data across groups?	Yes/No/Unclear
*Are missing outcome data imputed using appropriate methods?	Yes/No/Unclear

9) Are reports of the study free of selective outcome reporting? (*)

*Was the study protocol available and were all of the study's pre-specified primary and secondary outcomes reported in the current manuscript?	Yes/No/Unclear
*Was the study protocol not available, but was it clear that the published report included all expected outcomes (i.e. comparing methods and results section)?	Yes/No/Unclear

10) Was the study apparently free of other problems that could result in high risk of bias? (*)

*Was the study free of contamination (pooling drugs)?	Yes/No/Unclear
*Was the study free of inappropriate influence of funders?	Yes/No/Unclear
*Was the study free of unit of analysis errors?	Yes/No/Unclear
*Were design-specific risks of bias absent?	Yes/No/Unclear
*Were new animals added to the control and experimental groups to replace drop-outs from the original population?	Yes/No/Unclear

3. Xu 2019

1) Was the allocation sequence adequately generated and applied?

*Did the investigators describe a random component in the sequence generation process such as:

■ Referring to a random number table; Yes/No/Unclear

■ Using a computer random number generator.

Additional info:

2) Were the groups similar at baseline or were they adjusted for confounders in the analysis?

*Was the distribution of relevant baseline characteristics balanced for the intervention and control groups? Yes/No/Unclear

*If relevant, did the investigators adequately adjust for unequal distribution of some relevant baseline characteristics in the analysis? Yes/No/Unclear

*Was the timing of disease induction adequate? Yes/No/Unclear

3) Was the allocation to the different groups adequately concealed during?

*Could the investigator allocating the animals to intervention or control group not foresee assignment due to one of the following or equivalent methods? Yes/No/Unclear

4) Were the animals randomly housed during the experiment?

*Did the authors randomly place the cages or animals within the animal room/facility?

■ Animals were selected at random during outcome assessment (use signaling questions of entry 6). Yes/No/Unclear

*Is it unlikely that the outcome or the outcome measurement was influenced by not randomly housing the animals?

The animals from the various experimental groups live together in one cage/pasture (e.g., housing conditions are identical). Yes/No/Unclear

5) Were the caregivers and/or investigators blinded from knowledge which intervention each animal received during the experiment?

*Was blinding of caregivers and investigators ensured, and was it unlikely that their blinding could have been broken?

■ ID cards of individual animals, or cage/animal labels are coded and identical in appearance.

■ Sequentially numbered drug containers are identical in appearance. Yes/No/Unclear

■ The circumstances during the intervention are specified and similar in both groups (#).

■ Housing conditions of the animals during the experiment are randomized within the room (use criteria of entry 4).

6) Were animals selected at random for outcome assessment?

*Did the investigators randomly pick an animal during outcome assessment, or did they use a random component in the sequence generation for outcome assessment? Yes/No/Unclear

■ Referring to a random number table;

■ Using a computer random number generator;

■ Etc.

7) Was the outcome assessor blinded?

*Was blinding of the outcome assessor ensured, and was it unlikely that blinding could have been broken?	
■ Outcome assessment methods were the same in both groups.	Yes/No/Unclear
■ Animals were selected at random during outcome assessment (use signaling questions of entry 6).	
*Was the outcome assessor not blinded, but do review authors judge that the outcome is not likely to be influenced by lack of blinding? (e.g., mortality)	Yes/No/Unclear

8) Were incomplete outcome data adequately addressed? (*)

*Were all animals included in the analysis?	Yes/No/Unclear
*Were the reasons for missing outcome data unlikely to be related to true outcome? (e.g., technical failure)	Yes/No/Unclear
*Are missing outcome data balanced in numbers across intervention groups, with similar reasons for missing data across groups?	Yes/No/Unclear
*Are missing outcome data imputed using appropriate methods?	Yes/No/Unclear

9) Are reports of the study free of selective outcome reporting? (*)

*Was the study protocol available and were all of the study's pre-specified primary and secondary outcomes reported in the current manuscript?	Yes/No/Unclear
*Was the study protocol not available, but was it clear that the published report included all expected outcomes (i.e. comparing methods and results section)?	Yes/No/Unclear

10) Was the study apparently free of other problems that could result in high risk of bias? (*)

*Was the study free of contamination (pooling drugs)?	Yes/No/Unclear
*Was the study free of inappropriate influence of funders?	Yes/No/Unclear
*Was the study free of unit of analysis errors?	Yes/No/Unclear
*Were design-specific risks of bias absent?	Yes/No/Unclear
*Were new animals added to the control and experimental groups to replace drop-outs from the original population?	Yes/No/Unclear

Domain	Almeida, 2016		Andries, 2014		Xu, 2019	
	Answer	RoB*	Answer	RoB*	Answer	RoB*
Was the allocation sequence adequately generated and applied?	Yes	low	Unclear	Unclear	Yes	Low
Were the groups similar at baseline or were they adjusted for confounders in the analysis?	Yes	Low	Unclear	Unclear	Yes	Low
Was the allocation adequately concealed?	Unclear	Unclear	Unclear	Unclear	Unclear	Unclear
Were the animals randomly housed during the experiment?	Unclear	Unclear	Unclear	Unclear	Unclear	Unclear
Were the caregivers and /or investigators blinded from knowledge which intervention each animal received during the experiment?	Unclear	Unclear	Unclear	Unclear	Unclear	Unclear
Were animals selected at random for outcome assessment?	Yes	low	Unclear	Unclear	Yes	low
Was the outcome assessor blinded?	Unclear	Unclear	Unclear	Unclear	Unclear	Unclear
Were incomplete outcome data adequately addressed?	Yes	low	Unclear	Unclear	Unclear	Unclear

**RoB = Risk of Bias*

Supplement 7 – Geographical sample origin

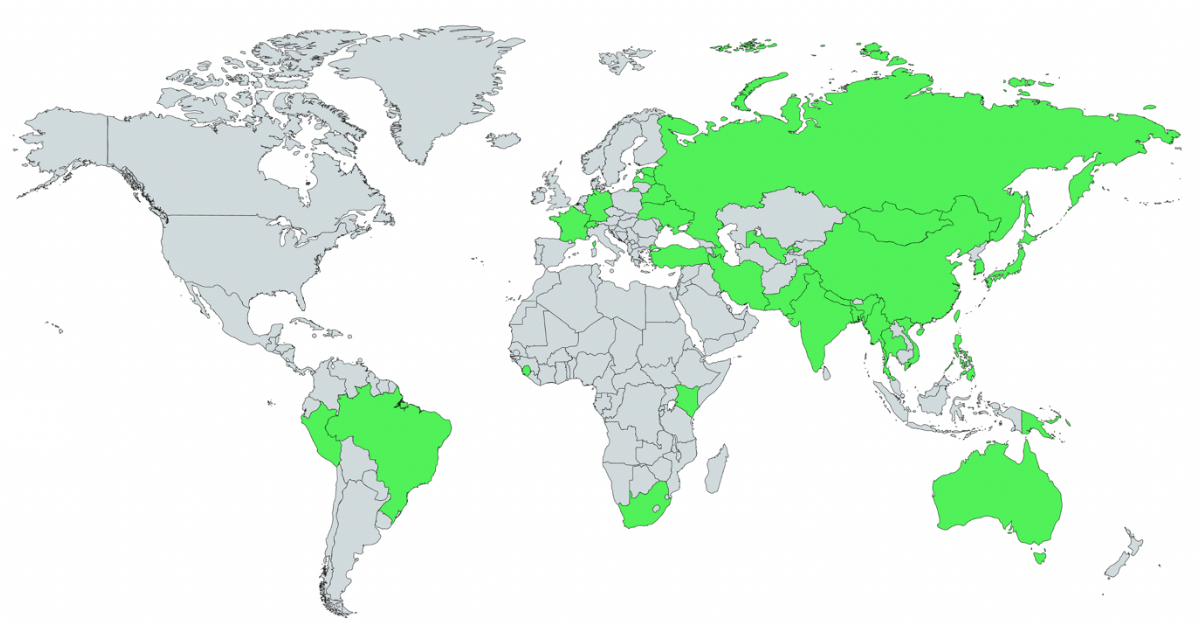

Clinical sample origin as indicated by authors in included studies.

Supplement 8 – pDST agreement

	MGIT (n=933)	Thermo (n=423)	7H11 (n=913)	7H10 (n=42)	MABA (n=70)	REMA (n=158)
MGIT	1	0.99 (393/396)	0.97 (445/459)			0 (0/4)
Thermo	0.99 (393/396)	1	1 (5/5)			
7H11	0.97 (445/459)	1 (5/5)	1			0.5 (2/4)
7H10				1	1 (2/2)	1 (2/2)
MABA				1 (2/2)	1	1 (2/2)
REMA	0 (0/4)		0.5 (2/4)	1 (2/2)	1 (2/2)	1

pDST agreement matrix based on isolates for which MIC data was available on multiple pDST platforms. High agreement (>0.95) is shown in green, low agreement (<0.95) is shown in red.

Supplement 9 – MIC 7H11

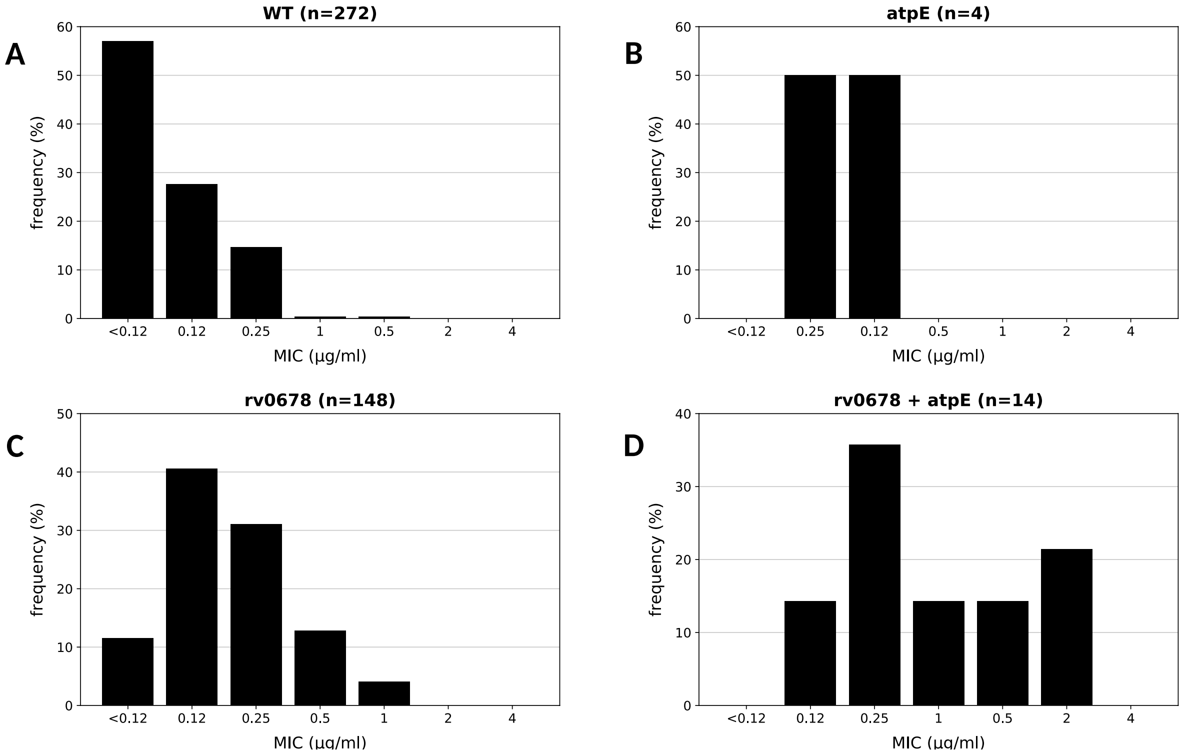

7H11 MIC distribution. Only isolates with information on both *atpE* and *rv0678* were included. Isolates of which the reported MIC could not be reported as one of the concentrations in this figure were excluded. (**2a**) 7H11 MIC distribution of wild type samples. (**2b**) 7H11 MIC distribution of isolates with one or more *atpE* variant and wild type *rv0678*. (**2c**) 7H11 MIC distribution of isolates with one or more *rv0678* variant and wild type *atpE*. (**2d)** 7H11 MIC distribution of isolates with one or more *atpE* and one or more *rv0678* variant.

Supplement 10 – MIC Thermo Fisher microtiter plate

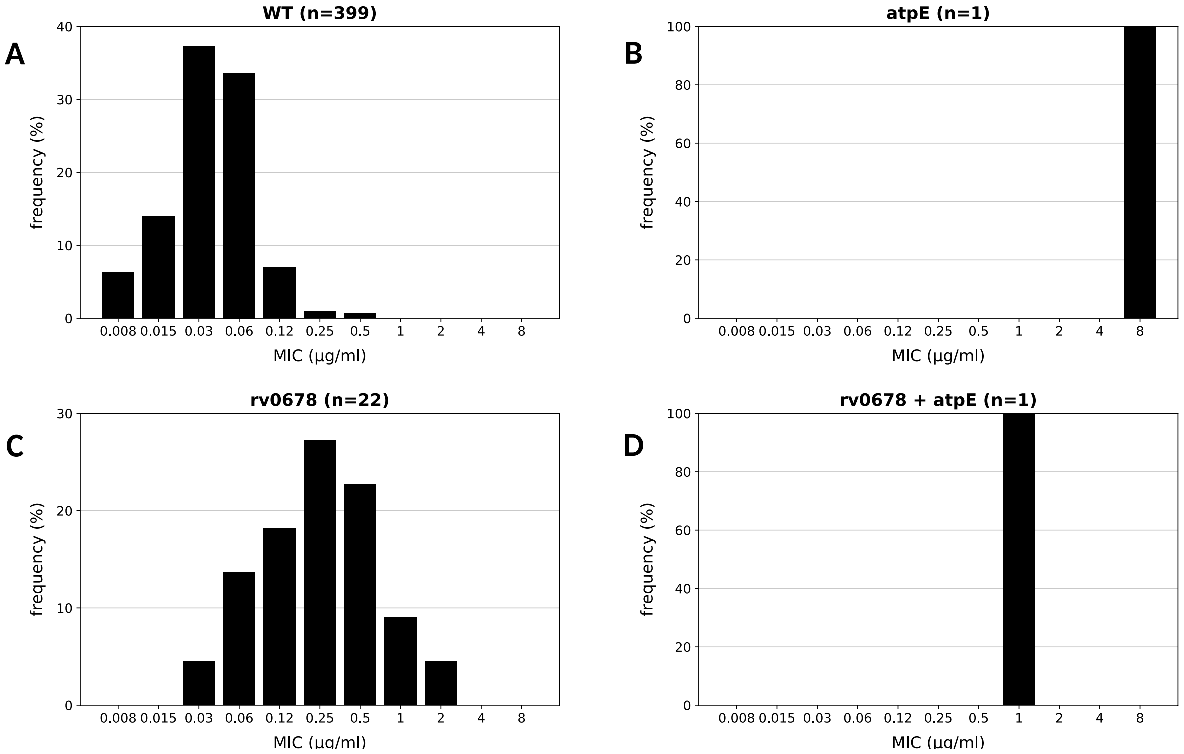

Thermo Fisher microplate MIC distribution. Only isolates with information on both *atpE* and *rv0678* were included. Isolates of which the reported MIC could not be reported as one of the concentrations in this figure were excluded. (**2a**) Thermo Fisher microplate MIC distribution of wild type samples. (**2b**) Thermo Fisher microplate MIC distribution of isolates with one or more *atpE* variant and wild type *rv0678*. (**2c**) Thermo Fisher microplate MIC distribution of isolates with one or more *rv0678* variant and wild type *atpE*. (**2d)** Thermo Fisher microplate MIC distribution of isolates with one or more *atpE* and one or more *rv0678* variant.

Supplement 11 – Rv0678 lollipop plot

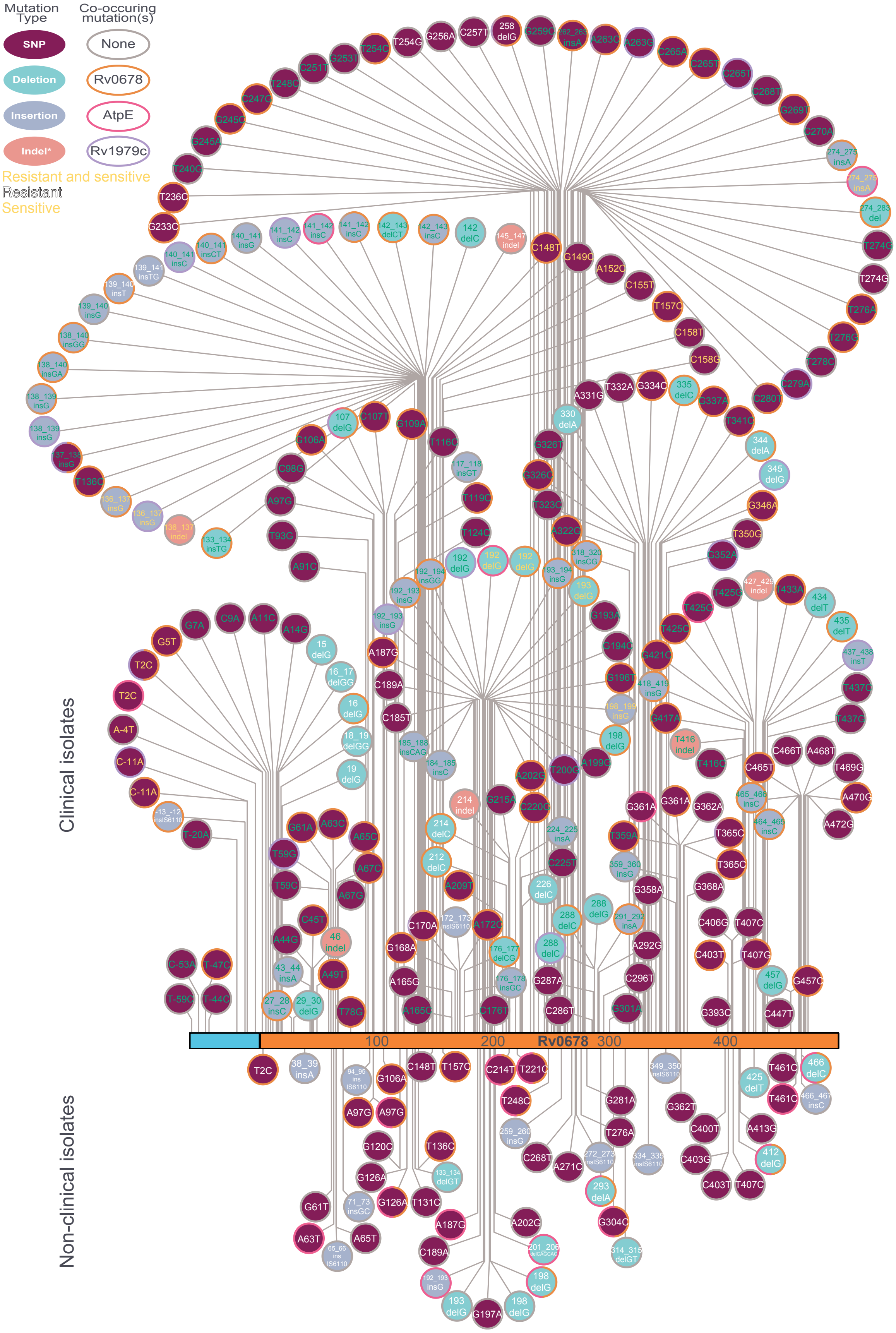

Lollipop figure with all observed variants across the *Rv0678* gene.

Supplement 12 – atpE lollipop plot

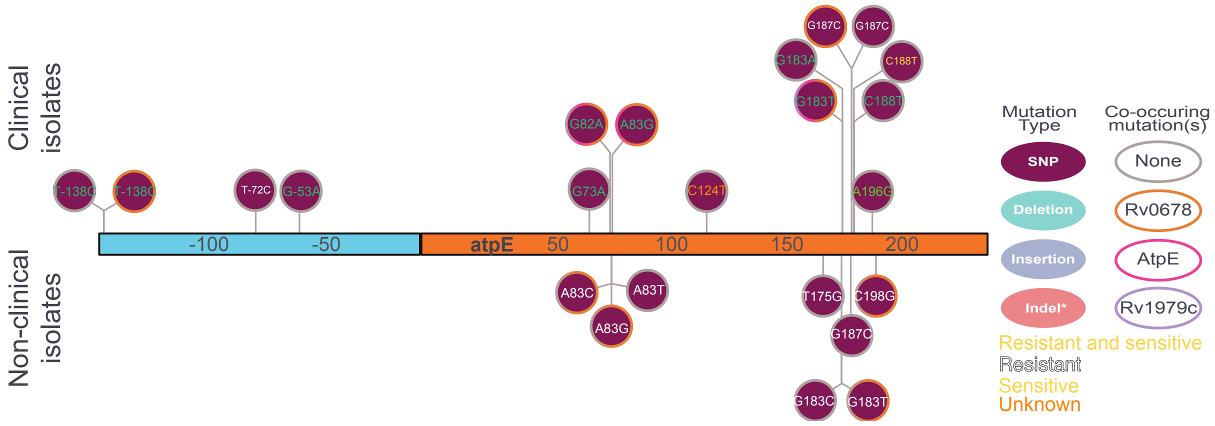

Lollipop figure with all observed variants across the *atpE* gene.

Supplement 13 – pepQ heatmap

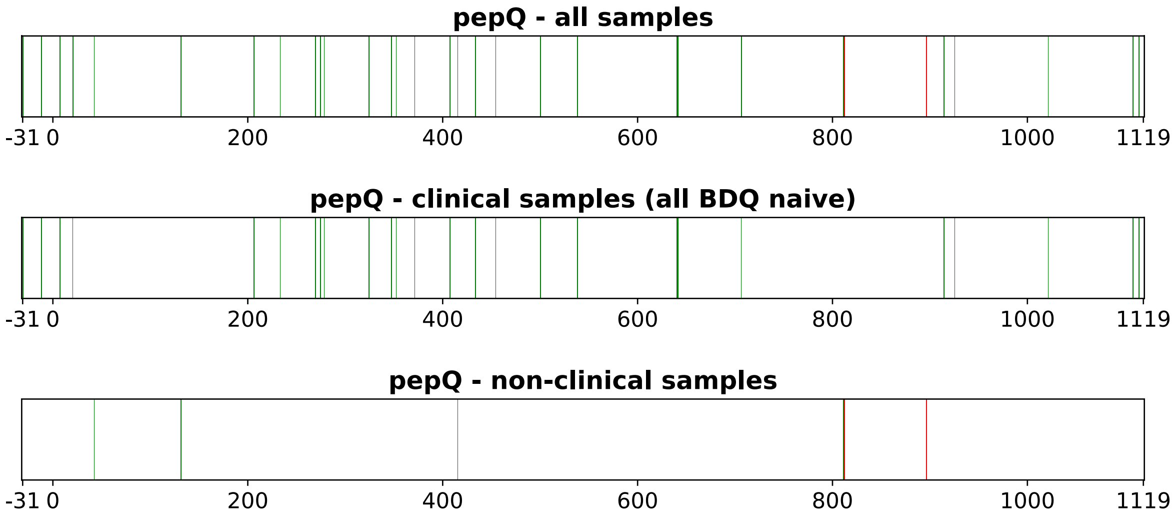

Heatmap of observed variants across the *pepQ* gene. Additional heatmaps stratified by isolate type are shown. All pepQ variants in clinical samples were observed in BDQ treatment naïve isolates. Green bars indicate that variants on those positions were exclusively observed in BDQ susceptible isolates. Red bars indicate that variants on those positions were exclusively observed in BDQ resistant isolates. Yellow bars indicate that variants on those positions were observed in a mix of BDQ susceptible and resistant isolates. White indicates no information on variants on those positions.

Supplement 14 – pepQ lollipop plot

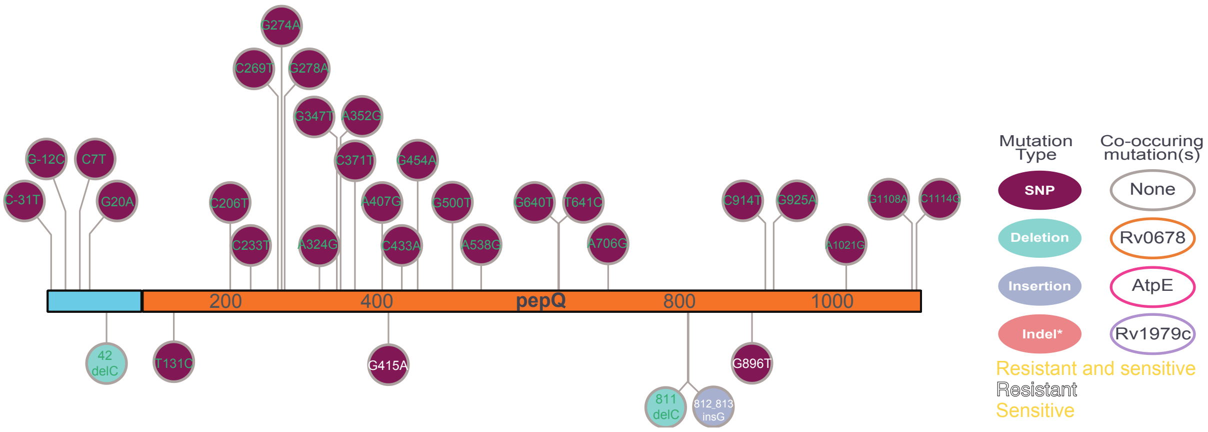

Lollipop figure with all observed variants across the *pepQ* gene.

Supplement 15 – Rv1979c heatmap

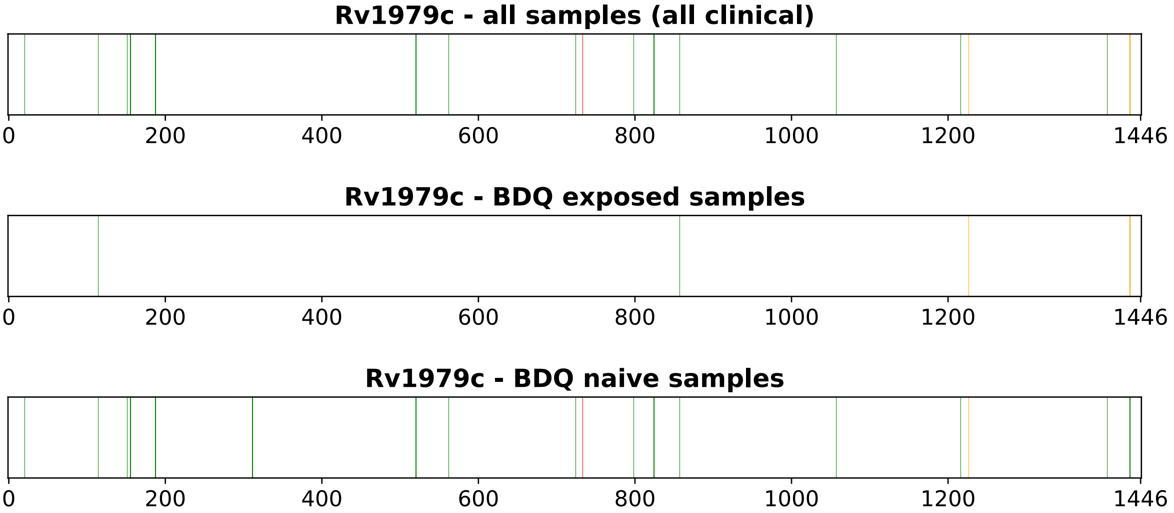

Heatmap of observed variants across the *Rv1979c* gene. Additional heatmaps stratified by BDQ treatment are shown. All *Rv1979c* variants were observed in clinical isolates. Green bars indicate that variants on those positions were exclusively observed in BDQ susceptible isolates. Red bars indicate that variants on those positions were exclusively observed in BDQ resistant isolates. Yellow bars indicate that variants on those positions were observed in a mix of BDQ susceptible and resistant isolates. White indicates no information on variants on those positions.

Supplement 16 – Rv1979c lollipop plot

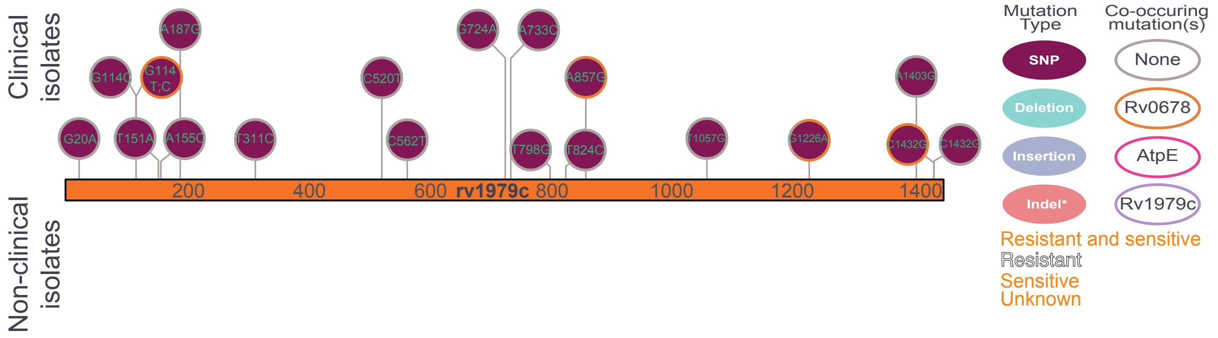

Lollipop figure with all observed variants across the *Rv1979c* gene.

Supplement 17 – Association between combinations of variants in the *atpE*, *Rv0678*, *pepQ*, and *Rv1979c* gene and phenotypic resistance

	Phenotypic DST results						Statistical association between phenotype and genetic variant$			
	All		Clinical		Non-clinical					
Combination of variants#	R	S	R	S	R	S	OR estimate	OR 95% CI lower bound	OR 95% CI upper bound	p-value**
[atpE] G187C + [Rv0678] 141_142insC	3	0	3	0	0	0	∞	3.355	∞	0.011
[atpE] A83C + [Rv0678] 201_206delCAGCAC	2	0	0	0	2	0	∞	1.514	∞	0.024
[atpE] G82A + [atpE] G183T	2	0	2	0	0	0	∞	1.514	∞	0.024
[Rv0678] 136_137insG + [Rv1979c] G1226A	2	0	2	0	0	0	∞	1.514	∞	0.024
[Rv0678] 138_139insG + [Rv0678] 141_142insC	2	0	2	0	0	0	∞	1.514	∞	0.024
[Rv0678] 141_142insC + [Rv0678] A322G	2	0	2	0	0	0	∞	1.514	∞	0.024
[atpE] C188T + [Rv0678] T425G	5	1	5	1	0	0	42.181	4.611	1972.953	0.0013
[Rv0678] 141_142insC + [Rv1979c] G1226A	3	1	3	1	0	0	24.781	1.953	1291.325	0.024
[Rv0678] 136_137insG + [Rv0678] T236C	0	2	0	2	0	0	0	0	42.847	1.0
[Rv0678] 140_141insC + [Rv1979c] G114T/C	0	2	0	2	0	0	0	0	42.847	1.0
[Rv0678] 141_142insC + [Rv0678] 192_193insG	0	2	0	2	0	0	0	0	42.847	1.0
[Rv0678] 192_193insG + [Rv0678] G106A	0	2	0	2	0	0	0	0	42.847	1.0
[Rv0678] 193delG + [Rv0678] 274_275insA	0	2	0	2	0	0	0	0	42.847	1.0
[Rv0678] G326T + [Rv0678] 464_465insC	0	2	0	2	0	0	0	0	42.847	1.0
[Rv0678] 288delC + [Rv0678] T119C + [Rv0678] 184_185insC	0	3	0	3	0	0	0	0	19.520	1.0
[Rv0678] A67C + [Rv0678] 418_419insG	0	3	0	3	0	0	0	0	19.520	1.0
Total	21	20	19	20	2	0				

The table is sorted on the OR estimate and within identical ORs on the OR 95% CI interval. This way, variant combinations that are observed only in resistant isolates are shown on top, while variant combinations that are observed only in susceptible isolates are shown on the bottom of the table.

R: Resistant; S: Susceptible

to be included studies had to report at a minimum on variants in both the *Rv0678* and *atpE* gene

$ statistical analysis using the standardized method published by Miotto et al.(1)

**p value adjusted for false discovery rate

Supplement References

1. Miotto P, Tessema B, Tagliani E, Chindelevitch L, Starks AM, Emerson C, et al. A standardised method for interpreting the association between mutations and phenotypic drug resistance in Mycobacterium tuberculosis. Eur Respir J. 2017;50(6).
2. World Health Organization. Technical report on critical concentrations for TB drug susceptibility testing of medicines used in the treatment of drug-resistant TB. 2018.
3. European Committee on Antimicrobial Susceptibility Testing. Breakpoint tables for interpretation of MICs and zone diameters Version 10.0. 2020.
4. Kaniga K, Aono A, Borroni E, Cirillo DM, Desmaretz C, Hasan R, et al. Validation of Bedaquiline Phenotypic Drug Susceptibility Testing Methods and Breakpoints: a Multilaboratory, Multicountry Study. J Clin Microbiol. 2020;58(4).
5. Yang JS, Kim KJ, Choi H, Lee SH. Delamanid, Bedaquiline, and Linezolid Minimum Inhibitory Concentration Distributions and Resistance-related Gene Mutations in Multidrug-resistant and Extensively Drug-resistant Tuberculosis in Korea. Ann Lab Med. 2018;38(6):563-8.
6. Yoshiyama T, Mitarai S, Takaki A, Aono A, Okumura M, Ohta K, et al. Multi-drug resistant tuberculosis with simultaneously acquired-drug resistance to bedaquiline and delamanid. Clin Infect Dis. 2020.
7. Koser CU, Cirillo DM, Miotto P. How To Optimally Combine Genotypic and Phenotypic Drug Susceptibility Testing Methods for Pyrazinamide. Antimicrob Agents Chemother. 2020;64(9).
8. Bloemberg GV, Keller PM, Stucki D, Trauner A, Borrell S, Latshang T, et al. Acquired Resistance to Bedaquiline and Delamanid in Therapy for Tuberculosis. N Engl J Med. 2015;373(20):1986-8.
9. Hoffmann H, Kohl TA, Hofmann-Thiel S, Merker M, Beckert P, Jaton K, et al. Delamanid and Bedaquiline Resistance in Mycobacterium tuberculosis Ancestral Beijing Genotype Causing Extensively Drug-Resistant Tuberculosis in a Tibetan Refugee. Am J Respir Crit Care Med. 2016;193(3):337-40.
10. Xu J, Tasneen R, Peloquin CA, Almeida DV, Li SY, Barnes-Boyle K, et al. Verapamil Increases the Bioavailability and Efficacy of Bedaquiline but Not Clofazimine in a Murine Model of Tuberculosis. Antimicrob Agents Chemother. 2018;62(1).
11. Veziris N, Bernard C, Guglielmetti L, Le Du D, Marigot-Outtandy D, Jaspard M, et al. Rapid emergence of Mycobacterium tuberculosis bedaquiline resistance: lessons to avoid repeating past errors. Eur Respir J. 2017;49(3).
12. Villellas C, Coeck N, Meehan CJ, Lounis N, de Jong B, Rigouts L, et al. Unexpected high prevalence of resistance-associated Rv0678 variants in MDR-TB patients without documented prior use of clofazimine or bedaquiline. J Antimicrob Chemother. 2017;72(3):684-90.
13. Zimenkov DV, Nosova EY, Kulagina EV, Antonova OV, Arslanbaeva LR, Isakova AI, et al. Examination of bedaquiline- and linezolid-resistant Mycobacterium tuberculosis isolates from the Moscow region. J Antimicrob Chemother. 2017;72(7):1901-6.
14. Pang Y, Zong Z, Huo F, Jing W, Ma Y, Dong L, et al. In Vitro Drug Susceptibility of Bedaquiline, Delamanid, Linezolid, Clofazimine, Moxifloxacin, and Gatifloxacin against Extensively Drug-Resistant Tuberculosis in Beijing, China. Antimicrob Agents Chemother. 2017;61(10).

15. Ismail NA, Omar SV, Joseph L, Govender N, Blows L, Ismail F, et al. Defining Bedaquiline Susceptibility, Resistance, Cross-Resistance and Associated Genetic Determinants: A Retrospective Cohort Study. EBioMedicine. 2018;28:136-42.
16. Martinez E, Hennessy D, Jelfs P, Crighton T, Chen SC, Sintchenko V. Mutations associated with in vitro resistance to bedaquiline in Mycobacterium tuberculosis isolates in Australia. Tuberculosis (Edinb). 2018;111:31-4.
17. Ghodousi A, Rizvi AH, Baloch AQ, Ghafoor A, Khanzada FM, Qadir M, et al. Acquisition of Cross-Resistance to Bedaquiline and Clofazimine following Treatment for Tuberculosis in Pakistan. Antimicrob Agents Chemother. 2019;63(9).
18. Ghajavand H, Kargarpour Kamakoli M, Khanipour S, Pourazar Dizaji S, Masoumi M, Rahimi Jamnani F, et al. High Prevalence of Bedaquiline Resistance in Treatment-Naive Tuberculosis Patients and Verapamil Effectiveness. Antimicrob Agents Chemother. 2019;63(3).
19. de Vos M, Ley SD, Wiggins KB, Derendinger B, Dippenaar A, Grobbelaar M, et al. Bedaquiline Microheteroresistance after Cessation of Tuberculosis Treatment. N Engl J Med. 2019;380(22):2178-80.
20. Polsfuss S, Hofmann-Thiel S, Merker M, Krieger D, Niemann S, Russmann H, et al. Emergence of Low-level Delamanid and Bedaquiline Resistance During Extremely Drug-resistant Tuberculosis Treatment. Clin Infect Dis. 2019;69(7):1229-31.
21. Rancoita PMV, Cugnata F, Gibertoni Cruz AL, Borroni E, Hoosdally SJ, Walker TM, et al. Validating a 14-Drug Microtiter Plate Containing Bedaquiline and Delamanid for Large-Scale Research Susceptibility Testing of Mycobacterium tuberculosis. Antimicrob Agents Chemother. 2018;62(9).
22. Xu J, Wang B, Hu M, Huo F, Guo S, Jing W, et al. Primary Clofazimine and Bedaquiline Resistance among Isolates from Patients with Multidrug-Resistant Tuberculosis. Antimicrob Agents Chemother. 2017;61(6).
23. Klopper M, Heupink TH, Hill-Cawthorne G, Streicher EM, Dippenaar A, de Vos M, et al. A landscape of genomic alterations at the root of a near-untreatable tuberculosis epidemic. BMC Med. 2020;18(1):24.
24. Nimmo C, Millard J, Brien K, Moodley S, van Dorp L, Lutchminarain K, et al. Bedaquiline resistance in drug-resistant tuberculosis HIV co-infected patients. Eur Respir J. 2020;55(6).
25. Andres S, Merker M, Heyckendorf J, Kalsdorf B, Rumetshofer R, Indra A, et al. Bedaquiline-Resistant Tuberculosis: Dark Clouds on the Horizon. Am J Respir Crit Care Med. 2020;201(12):1564-8.
26. Peretokina IV, Krylova LY, Antonova OV, Kholina MS, Kulagina EV, Nosova EY, et al. Reduced susceptibility and resistance to bedaquiline in clinical M. tuberculosis isolates. J Infect. 2020;80(5):527-35.
27. Conradie F, Diacon AH, Ngubane N, Howell P, Everitt D, Crook AM, et al. Treatment of Highly Drug-Resistant Pulmonary Tuberculosis. N Engl J Med. 2020;382(10):893-902.
28. Battaglia S, Spitaleri A, Cabibbe AM, Meehan CJ, Utpatel C, Ismail N, et al. Characterization of Genomic Variants Associated with Resistance to Bedaquiline and Delamanid in Naive Mycobacterium tuberculosis Clinical Strains. J Clin Microbiol. 2020;58(11).
29. Torrea G, Coeck N, Desmaretz C, Van De Parre T, Van Poucke T, Lounis N, et al. Bedaquiline susceptibility testing of Mycobacterium tuberculosis in an automated liquid culture system. J Antimicrob Chemother. 2015;70(8):2300-5.

30. World Health Organization. The use of next-generation sequencing technologies for the detection of mutations associated with drug resistance in Mycobacterium tuberculosiscomplex: technical guide. 2018.
31. Nimmo C, Millard J, van Dorp L, Brien K, Moodley S, Wolf A, et al. Population-level emergence of bedaquiline and clofazimine resistance-associated variants among patients with drug-resistant tuberculosis in southern Africa: a phenotypic and phylogenetic analysis. Lancet Microbe. 2020;1(4):e165-e74.
32. Liu Y, Gao M, Du J, Wang L, Gao J, Shu W, et al. Reduced susceptibility of Mycobacterium tuberculosis to bedaquiline during antituberculosis treatment and its correlation with clinical outcomes in China. Clin Infect Dis. 2020.
33. Yang J, Pang Y, Zhang T, Xian X, Li Y, Wang R, et al. Molecular characteristics and in vitro susceptibility to bedaquiline of Mycobacterium tuberculosis isolates circulating in Shaanxi, China. Int J Infect Dis. 2020;99:163-70.
